# 3-D shallow shear velocity structure of the Jakarta Basin from transdimensional ambient noise tomography

**DOI:** 10.1093/gji/ggad176

**Published:** 2023-05-03

**Authors:** Rexha Verdhora Ry, Phil R Cummins, Babak Hejrani, Sri Widiyantoro

**Affiliations:** Research School of Earth Sciences, The Australian National University, Canberra ACT 2601, Australia; Global Geophysics Research Group, Faculty of Mining and Petroleum Engineering, Institut Teknologi Bandung, Bandung 40132, Indonesia; Research School of Earth Sciences, The Australian National University, Canberra ACT 2601, Australia; Geoscience Australia, Canberra ACT 2609, Australia; Research School of Earth Sciences, The Australian National University, Canberra ACT 2601, Australia; Geoscience Australia, Canberra ACT 2609, Australia; Global Geophysics Research Group, Faculty of Mining and Petroleum Engineering, Institut Teknologi Bandung, Bandung 40132, Indonesia; Faculty of Engineering, Maranatha Christian University, Bandung 40164, Indonesia

**Keywords:** Asia, Seismic interferometry, Seismic tomography, Site effects, Surface waves and free oscillations

## Abstract

Situated on the northern coast of the Indonesian island of Java, Jakarta and its metropolitan area (Greater Jakarta) are subject to significant earthquake hazards from a subduction zone south of Java and nearby active crustal faults. The seismic risk may be even higher because Greater Jakarta resides on a sedimentary basin filled with thick Pliocene–Pleistocene sediments. A comprehensive study of Jakarta Basin's properties and geometry is important for creating robust seismic hazard and risk assessments. The main objective of this study is to develop a 3-D model of Jakarta Basin's shallow shear-wave velocity (*V_S_* ) structure and improve on previous models that did not cover the basin edge due to the extent of data coverage. Between April and October 2018, we deployed a new temporary seismic network to extend the spatial coverage beyond that of a previous deployment in 2013, and sampled 143 locations through sequential deployments of 30 broad-band sensors covering Jakarta and its adjacent areas. We conducted a 2-stage transdimensional Bayesian inversion of Rayleigh wave phase velocity dispersion curves derived from seismic noise. To begin, we applied tomography and constructed 2-D phase velocity maps for periods 1–5 s. Then, at each point in a regular grid defined on these maps, we invert each dispersion curve into 1-D depth profiles of *V_S_* . Finally, these profiles at gridpoints with ∼2 km spacing are interpolated to form a pseudo-3-D *V_S_* model. Our results reveal the edge of the Pliocene–Pleistocene sediments along the south. Also, we resolve a basement offset across south Jakarta that we suggest may be related to the western extension of the Baribis Fault (alternatively, the West Java Backarc Thrust). We recommend using this 3-D model of the Jakarta Basin for scenario earthquake ground motion simulations. Such simulations would help establish how important it might be to re-assess seismic hazard and risk in Greater Jakarta so that basin resonance and amplification are considered.

## 1. INTRODUCTION

The city of Jakarta, Indonesia's capital and one of the world's megacities, is located on the northern coast of the Indonesian island of Java. Greater Jakarta (Jakarta and its metropolitan area: Depok, Tangerang, Bekasi and Bogor) is one of the world's most populous urban areas (United Nations 2018) with about 35 million inhabitants in an area of 3367 km^2^, while more than 11 million residents live within Jakarta itself. The region is subject to significant earthquake hazards from a subduction zone south of Java and active crustal faults in West Java. In consequence, Jakarta's high population density of more than 14 000 people per square km means the city is likely a hotspot of seismic risk.

**Figure 1.**
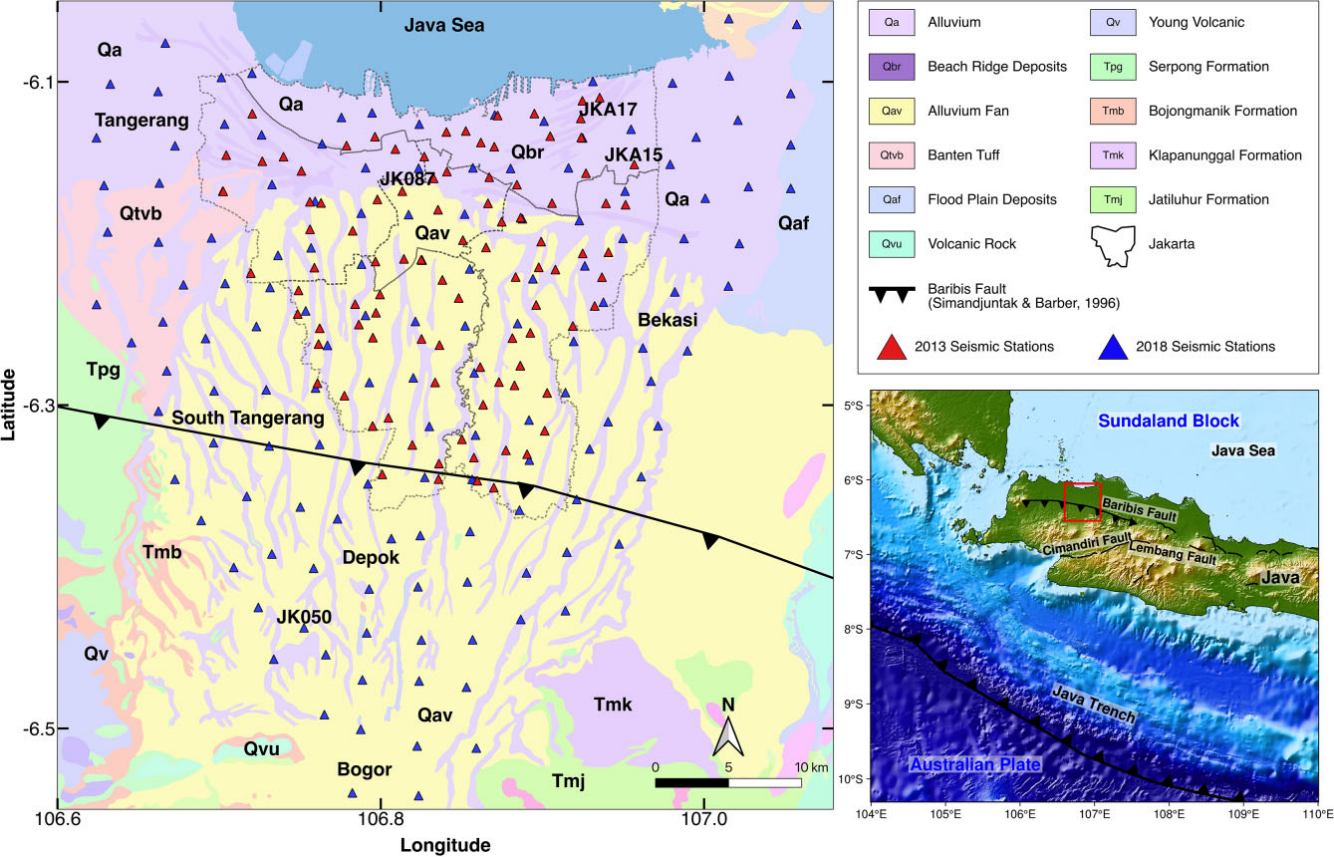
Map of the seismic deployments and surface geology around Jakarta, modified from the regional geological maps (Turkandi *et al*. 1992; Simandjuntak & Barber 1996; Effendi *et al*. 1998). Red triangles are the seismic stations deployed between October 2013 and February 2014. Blue triangles are the seismic stations deployed between April 2018 and October 2018. The province and subprovince administrative boundaries of Jakarta are shown with dashed lines.

At the Java Trench (see Fig. 1), about 300 km south of Jakarta, the Australian Plate subducts beneath the Sundaland Block with a convergence rate of about 60 mm yr^−1^ (Simons *et al*. 2007; Koulali *et al*. 2017). The closest segments of this subduction megathrust to Jakarta are Selat Sunda and West-Central Java, which have the potential for generating earthquakes as large as magnitude 8.7 (Irsyam *et al*. 2020). In addition, Jakarta and its metropolitan area are at risk from earthquakes that may occur on active crustal faults, namely the Cimandiri (Katili & Soetadi 1971), Lembang (Daryono *et al*. 2019) and Baribis (Simandjuntak & Barber 1996; Koulali *et al*. 2017; Damanik *et al*. 2021) Faults. The Baribis Fault, a backarc thrust fault in West Java, has been active since the Pliocene but is less clearly expressed in its western extent, near Jakarta, than it is farther east (Aribowo *et al*. 2022). Subsequently, Widiyantoro *et al*. (2022) also denote that the western Baribis Fault running through the southern part of Jakarta is locked and may accumulate elastic stress energy.

The seismic risk is enhanced by Jakarta's location on a sedimentary basin filled with geologically young sediments. Jakarta and its vicinity's surface geology are composed almost entirely of alluvium and beach ridge deposits (Turkandi *et al*. 1992). Pliocene–Pleistocene sediments of the Jakarta Basin gradually thicken from its southern edge with a thickness of around 50 m to central Jakarta with a thickness of more than 350 m (Fachri *et al*. 2002; Lubis *et al*. 2008). Such a thick young sedimentary layer enclosed in a basin should experience resonance and amplification of seismic ground motion (e.g. Rial *et al*. 1992), as has been observed in the 1985 Michoacan (Kawase & Aki 1989), the 1995 Kobe (Kawase 1996), the 2015 Kathmandu (Galetzka *et al*. 2015) and the 2017 Mexico City (Sahakian *et al*. 2018) earthquakes. In these and other events, poorly consolidated sediments and basin geometries caused amplification and prolongation of shaking, which strongly influenced the type and extent of the damage.

A thorough study of a basin's seismic properties and geometry is crucial to creating robust seismic hazard and risk assessments. To our knowledge, seismic hazard assessments for Greater Jakarta (Irsyam *et al*. 2020) are still developing and have yet to consider sedimentary basin-related effects on earthquake's ground motion. Studies have shown the importance of 3-D geometry for modelling seismic wave propagation in basins due to edge-diffracted waves (Kawase 1996; Ewald *et al*. 2006) and local-scale multiscattering and prolonged ground motion (Olsen *et al*. 2006; Denolle *et al*. 2014; Cruz-Atienza *et al*. 2016).

Such basin geometry can be modelled by constraining the velocity structure. The methods may include boreholes, active seismic surveys such as seismic reflection and refraction, or passive measurements such as microtremor and seismic interferometry. However, direct approaches are impractical and too expensive in urban areas, so the Jakarta Basin studies mainly rely on passive seismic (Ridwan *et al*. 2016, 2019; Saygin *et al*. 2016; 2017; Cipta *et al*. 2018a) and few borehole measurements (Fachri *et al*. 2002). These studies were limited to the city of Jakarta, yet the Jakarta Basin appears to extend beyond the city into Greater Jakarta.

This study aims to develop a 3-D model of Jakarta Basin's shallow shear-wave velocity (*V_S_*) structure. We propose a new model of the Jakarta Basin based on two-step transdimensional seismic ambient noise tomography; to improve previous models that have not revealed how far the basin extends beyond the city limits. The model we developed in this study shows the expansion of sedimentary basin fill that mainly reaches Greater Jakarta and the edge of Pliocene–Pleistocene sediments in the south. We also present an apparent basement offset across south Jakarta, which may be related to the western extension of the Baribis Fault.

## 2. THE JAKARTA BASIN

Fachri *et al*. (2002) used borehole data to establish that the Jakarta Basin stratigraphy from old to young mainly consists of formations from the Middle Miocene to the Pliocene–Holocene. Pleistocene–Holocene sediments dominate the near-surface geology of Jakarta and its vicinity, with thickness varying between 31.5 and 53 m (described as alluvium fan deposits in Turkandi *et al*. 1992). Pliocene sediment lies beneath with thickness ranging from 250 to 400 m and includes a mixture of sandstone and claystone. The Pliocene–Pleistocene sediments are found in most borehole logs in Greater Jakarta (Fachri *et al*. 2002). They form aquifer and aquitard zones in the Jakarta Basin hydrostratigraphy.

The Pliocene sediments are underlain by Miocene sediments that behave as a hydrological basement (Fachri *et al*. 2002; Lubis *et al*. 2008). The Miocene sediment may be regarded as engineering bedrock due to its higher compaction, although it is not necessarily a seismological basement. Evidence presented in boreholes (Fachri *et al*. 2002) exhibits different interfaces of Miocene sediments. Late Miocene sediments were found mainly in the north, while the south had mainly sediment of Middle Miocene age.

Aside from the few geological studies, the Jakarta Basin has been exposed to few passive geophysical measurements. The following studies focus on the area of Jakarta to estimate the basin thickness underneath the city. Ridwan *et al*. (2016) applied the spatial autocorrelation (SPAC) method and suggested that the depth of engineering bedrock (*V_S_* > 750 m s^−1^) reaches 725 m in the north and gradually decreases southward to about 350 m in the south. Saygin *et al*. (2016) developed a 3-D model for the basin structure by extracting Rayleigh wave group velocity measurements using Green's functions obtained from cross-correlograms of ambient noise at different station pairs. They suggested that the sedimentary basin covers most of the area of the city with a thickness of up to 1500 m below central Jakarta. The same data were subsequently used in studies reaching similar findings, in which *P*-wave reflectivity obtained from seismic noise was used to estimate basin depth (Saygin *et al*. 2017), and horizontal-to-vertical spectral ratio (HVSR) curves were used to estimate refined *V_S_* structure (Cipta *et al*. 2018a).

These previous studies have consistently shown a basin fill with average *V_S_* of about 500 m s^−1^ that increases in thickness (300–1000 m) from south to north. However, the extent of these deployments appears not to have covered the edge of the basin, so that estimates of basement depth obtained by these earlier studies were greater than 300–400 m, even at the southern edge of the array coverage. In this study, we attempted to resolve the basin edge by deploying a new seismic experiment comprising 143 points to cover Greater Jakarta, extending to outside the city limits of Jakarta itself.

## 3. DATA AND METHODS

This study combines two temporary seismic networks that were deployed separately: (1) the 2013–2014 network (see Saygin *et al*. 2016) and (2) the 2018 network. Both networks used three-component broad-band seismometers (Trillium Compact) and digitizers built by the Australian National University. The instruments were installed temporarily in schools, on concrete slab floors, throughout the city with a spacing of 3–5 km (Fig. 1). The 2013–2014 network covered most of the area within Jakarta's city limits with 96 stations. This network has been used in earlier studies to model the Jakarta Basin's geometry (Saygin *et al*. 2016; 2017; Cipta *et al*. 2018a). For this study, in 2018, we deployed another seismic network aiming mainly to expand the coverage outside Jakarta to reveal the extent of the sedimentary basin. This 2018 deployment comprised 143 stations where the 30 seismometers were maintained and redeployed in five phases with at least one month of recording at each site (Ry *et al*. 2019).

We used seismic ambient noise tomography (ANT) to the data of both seismic networks. The data analysis follows this workflow: (1) we derived the noise correlation function of Rayleigh wave from the cross-correlation of vertical seismograms, (2) we estimated the phase velocity dispersion curve at each interstation midpoint from the real part of the NCF's spectrum, (3) we invert these dispersion curves to map 2-D Rayleigh wave velocity tomograms at various periods and (4) we invert dispersion curves for *V_S_* depth profiles on a set of the regularly spaced gridpoints covering the study area and then interpolate these to obtain a pseudo-3-D *V_S_* model of the Jakarta Basin.

### 3.1. Noise correlation functions

To assess surface waves, the empirical Green's function (GF) between two stations is often derived by cross-correlating their background ambient noise (Shapiro & Campillo 2004). Various processing workflows for this have been suggested, such as in Yao *et al*. (2006), Bensen *et al*. (2007) and Seats *et al*. (2012). Bensen *et al*. (2007), suggest the calculation of daily seismogram cross-correlations (correlograms); then, the daily correlograms over weeks, months, or years are stacked together to enhance the emergence of the surface wave signal. These stacked correlograms are usually called a noise correlation function (NCF). Meanwhile, Seats *et al*. (2012) propose shorter and overlapping time windows for cross-correlation and stacking to enhance the signal in NCF. They note that this approach benefits ambient seismic studies with limited recording duration and high levels of discontinuous local noise.

In this study, we used the HiPerSeis package (Hassan *et al*. 2020) to obtain Rayleigh wave NCFs. The use of HiPerSeis, which is optimized for high-performance computing clusters and therefore allows for rapid assessment of processed results, allowed us to test many different choices for processing options and parameters (Hejrani *et al*. 2020). Prior to the calculation of NCFs, we checked the data for possible GPS clock drifts and other timing errors. Such problems are common in seismic instruments for various reasons and authors have taken different approaches to identify or correct for such errors (see, e.g. Hejrani *et al*. 2015, 2017, 2020; Hable *et al*. 2018). Using functionalities of HiPerSeis (Gorbatov *et al*. 2020), we assessed the GPS clock stability at a daily level for each station and we did not observe any drifts in the clocks.

We briefly describe our workflow to calculate NCFs as follows. The vertical seismograms for all stations in miniseed format were converted to ASDF. The data in the time domain was resampled to 10 Hz, then means and trends were removed. Bandpass filtering was performed between 0.02 and 4 Hz. The data was normalized in the time domain so that it is approximately distributed as a standard normal distribution (e.g. Hawkins & Sambridge 2019). Spectral whitening was applied with a window frequency of 0.02 Hz. The vertical seismograms for all available station pairs were cross-correlated in one-hour-long segments with 75 per cent overlap (Seats *et al*. 2012). The results were stacked linearly over the entire recording period.

**Figure 2.**
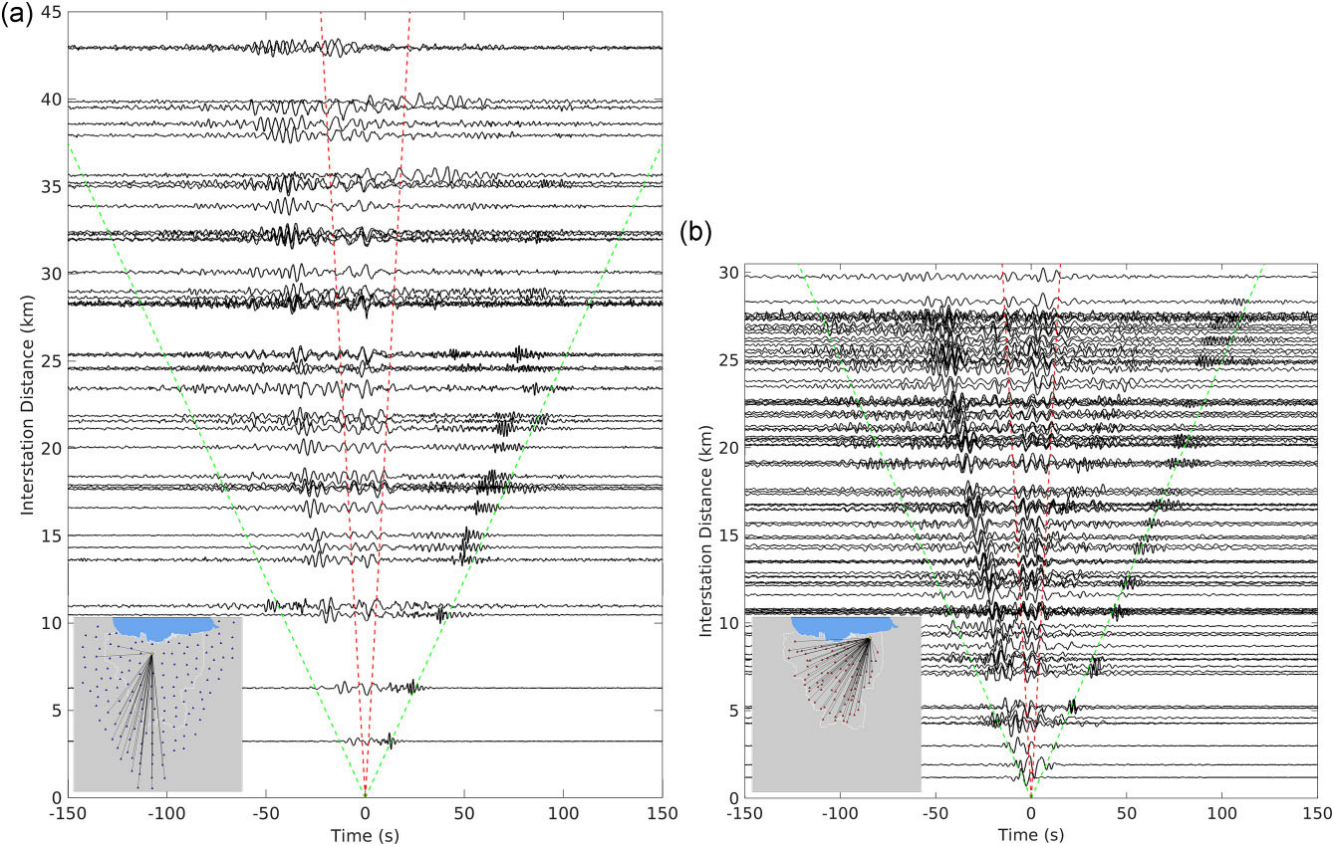
Retrieved Green's functions of Rayleigh waves between the reference stations: (a) JK087 (the 2018 network), (b) JKA17 (the 2013–2014 network) and others. Correlograms were filtered between 0.1 and 0.5 Hz for plotting. Red and green lines show group velocities of 2 and 0.25 km s^−1^, respectively. Inset map shows the location of the reference source stations (yellow stars) and receiver stations (triangles).

To assess the excitation of surface waves, we visualize two sets of NCFs in Fig. 2, one from our 2018 deployment (Fig. 2a) and the other from the 2013 to 2014 deployment (Fig. 2b). We term the waves propagating towards the reference station as causal and away from the station as acausal (the opposite convention to Saygin *et al*. 2016). It can be observed that the NCFs are asymmetrical and emerge more clearly in the acausal part of the correlograms. Generally, the NCF is a good approximation to GF when the noise sources are randomly but homogenously distributed (Wapenaar *et al*. 2005; Snieder 2007). However, in many real data applications, the background seismic energy is not evenly distributed as it depends on the coupling between ocean waves and coastlines. In this study area, the asymmetrical NCFs strongly suggest a non-homogenous distribution of the ambient noise sources at the frequencies of interest. The most robust energy appears in the acausal part of the NCFs of the north reference stations, indicating that the source of noise is dominantly in the north, that is an embayment of the Java Sea. Other studies of ambient noise in the central and western part of Java (Zulfakriza *et al*. 2014; Pranata *et al*. 2020; Rosalia *et al*. 2020; Yudistira *et al*. 2021) also illustrate this dominance of the ambient seismic noise wavefield by sources in the Java Sea.

Saygin *et al*. (2016) also noted the asymmetric character of the NCFs in the Jakarta Basin, particularly along paths with nearly SN orientation, and interpreted this asymmetry as reflecting fundamental mode propagation in the SN direction (causal using our convention in Fig. 2), and 1st order overtone propagation in the NS direction (acausal in Fig. 2), based on the higher group velocity of the latter. We had difficulty following this interpretation for two reasons: (1) The arrival in the acausal part of the NCF is always at least as pronounced as that in the causal part and (2) The arrival in the causal part of the NCF vanishes for paths that extend outside the basin (as seen in the upper right portion of Fig. 2a). While several other studies have noted the presence of overtones in NCFs recorded in sedimentary basins (Savage *et al*. 2013; Boué *et al*. 2016), and in some cases, the 1^st^ overtone can be dominant (Rivet *et al*. 2015), we know of no other case where fundamental and 1st overtone are so clearly separated into causal and acausal parts of the NCF due to anisotropic noise.

In developing an alternative interpretation, we note that the fundamental change in character between the causal and acausal parts of the Jakarta Basin NCFs is one of frequency content: the causal part (SN propagation in our convention) always involves higher frequencies than the acausal part. This is illustrated in Fig. S1, where we have used the same data from Fig 2(a) and displayed separately the spectra associated with the causal and acausal parts. The signal in the acausal part of the NCF is confined to frequencies below 0.4 Hz, while the causal part contains energy in the range 0.5–2 Hz. We speculate that, for reasons we have yet to ascertain, the constructive interference of the ambient noise to produce a GF is more effective for SN (causal) propagation in the higher frequency band (up to 2 Hz), and more effective for NS (acausal) propagation in the lower frequency band. The much lower apparent velocity of the causal arrival with respect to the acausal arrivals could simply be because the causal arrivals correspond to the slower, high-frequency component of the fundamental mode GF, while the acausal arrivals correspond to the faster, low-frequency component of the fundamental mode GF.

This interpretation is illustrated in Figs S2(a) and (b), where we compare synthetics that include only the fundamental mode with those that include both fundamental and 1st overtone, calculated for a *V_S_* profile near the centre of the stations used in the record section of Fig. S1. We follow Boué *et al*. (2016) in using a surface point source excitation (Harkrider & Anderson 1966) to calculate fundamental and 1st overtone spectra (Fig. S2c), and high-pass filter the causal part of the synthetics at 0.5 Hz, while the acausal part is low-passed filtered at 0.4 Hz. While the presence of the 1st overtone has some influence on the waveforms in both causal and acausal parts (Fig. S2b), the dominance of the fundamental mode is reflected in the similarity of waveforms in Figs S2(a) and (b), and in the spectra of Fig. S2(c). We also note that the phase velocity curves of fundamental and overtone are well separated, with no osculation. While we have only established self-consistency and not necessarily veracity of our interpretation that the acausal and causal parts of the NCF are both mainly associated with the fundamental mode, we believe this interpretation is as viable as the fundamental versus 1st overtone interpretation of Saygin *et al*. (2016). Therefore, we assume in what follows that both causal and acausal parts of our NCFs can be interpreted as reflecting the fundamental mode only. We leave the resolution of how much (or how little) the 1st overtone contributes to our NCFs to future work.

### 3.2. Estimation of phase velocity

We analysed the NCFs of all station pairs to estimate the phase velocity of Rayleigh waves. Aki (1957) established how the average cross-spectrum $\rho ( {r,{\omega }_0} )$ of isotropic ambient seismic noise varies with phase velocity according to the Bessel function of the first kind, ${J}_0$:

(1) \begin{eqnarray*}
\rho \left( {r,{\omega }_0} \right){\mathrm{\ }} = {\mathrm{\ \ }}{J}_0\left( {{\mathrm{\ }}\frac{{{\omega }_0}}{{c({\omega }_0)}}r{\mathrm{\ }}} \right),
\end{eqnarray*}

where $c({\omega }_0)$ is the phase velocity at frequency ${\omega }_0$ and *r* is the interstation distance.

Ekström *et al*. (2009) used eq. (1) for estimating phase velocity dispersion curves across arrays in the western United States. They derive eq. (1) to measure a dispersion curve but only consider fitting the real part of the cross-spectrum to the zeros of the Bessel function since it is less susceptible to background noise energy, as:

(2) \begin{eqnarray*}
c\left( {{\omega }_n} \right){\mathrm{\ }} = {\mathrm{\ \ }}\frac{{{\omega }_n}}{{{Z}_n}}r
\end{eqnarray*}

where ${\omega }_n$ is the frequency of the *n*^th^ observed zero crossing and ${Z}_n$ is the *n*th zero of the ${J}_0$ Bessel function. However, noise in the spectrum often causes missed or extra zeros. Hence, eq. (2) needs to adopt a range of phase velocities as:

(3) \begin{eqnarray*}
{c}_m\left( {{\omega }_n} \right){\mathrm{\ }} = {\mathrm{\ \ }}\frac{{{\omega }_n}}{{{Z}_{n + 2m}}}r
\end{eqnarray*}

where *m* is 0, ±1, ±2, …, pointing to the number of missed or extra zero crossings. Missed or extra zeros results in jumps or discontinuities in the range of phase velocities branches obtained from eq. (3) (Hejrani *et al*. 2020).

In consequence, Prieto *et al*. (2009) suggested a way to estimate an average phase velocity dispersion curve beneath an array. They demonstrated this by measuring the coherency in a frequency–distance domain between the real part of all NCFs spectrum and the Bessel function. Both approaches by Ekström *et al*. (2009) and Prieto *et al*. (2009) assess phase velocity from the relation between zero crossings of the NCF spectrum and the Bessel function. Accordingly, Hejrani *et al*. (2020). combined those to develop a two-stage semi-automatic routine to extract dispersion curves efficiently.

We measured phase velocity dispersion curves following Hejrani *et al*. (2020), who did this in two stages. First, an average dispersion curve was calculated beneath an array (or a subarray) following Prieto *et al*. (2009). Given that the shallow lithologies in our study area are relatively complex and we attempted to obtain dispersion curves at frequencies up to 1 Hz, we estimated multiple average dispersion curves for three subsets of stations (see Fig. S3). Secondly, dispersion curves were picked manually among the branches identified with eqs (2) and (3) (Ekström *et al*. 2009) following these rules: (1) to select the correct branch of dispersion curve among the ones generated in eq. (3) for various values of *m*, we used our average dispersion curve as a guide at low frequencies. We selected a branch that is within one cycle (up and down) of the reference dispersion curve. The lowest frequency we consider was defined by the longest wavelength that could be detected given the inter-station distance. (2) We then manually assessed the selected branch to higher frequencies, up to 1 Hz. We considered jumps and drops in the dispersion curves due to missing or extra zeros in the spectrum as well as the amplitude of the oscillations in the spectrum.

**Figure 3.**
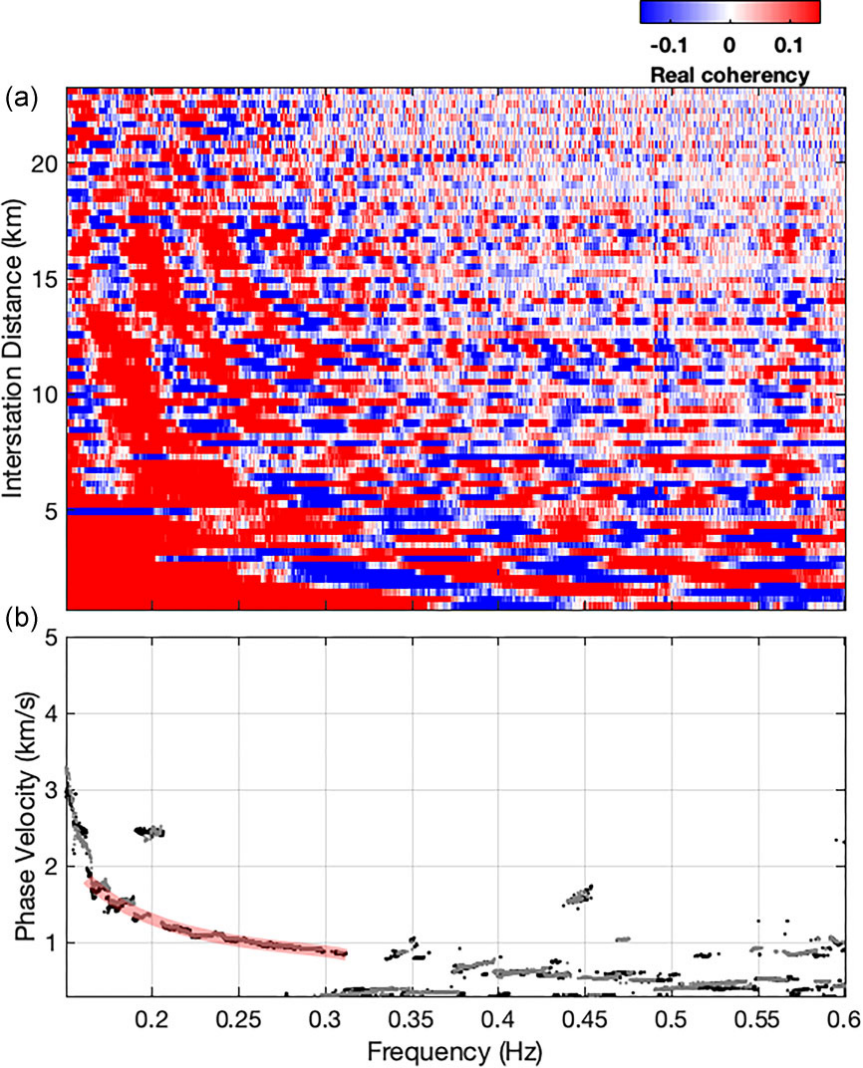
(a) Observed real part of NCFs spectrum binned in the frequency–distance domain. (b) Estimated average phase velocity dispersion curve by fitting the observed coherency and the Bessel function following Prieto *et al*. (2009). Dots show the best fits of phase velocity as a function of frequency. Light-red line emphasizes the average dispersion curve between 0.16 and 0.32 Hz.

To generate average dispersion curves, we considered three subsets of stations, named subset-1, subset-2 and all-pairs, where subsets 1 and 2 are sitting in different geological settings (see Fig. S3). Following Prieto *et al*. (2009), the station pairs in each subset were binned based on their interstation distance (250 meters bins), and the one with the highest SNR is selected in each bin (Fig. 3a). At a given frequency, the spectrum of NCFs in the frequency–distance domain are modelled using Bessel functions of the first kind, ${J}_0( {{\omega }_0,r} )$, by grid search over phase velocity. The result is an average dispersion curve for the selected station pairs (Fig. 3b). In the case of subset-1 (Figs S1b and S3), we observed stable phase velocities at 0.16–0.32 Hz to obtain the average dispersion curve depicted by the grey line. We could not extract an average dispersion curve at higher frequencies, probably due to the heterogeneity of the shallower crustal structure.

**Figure 4.**
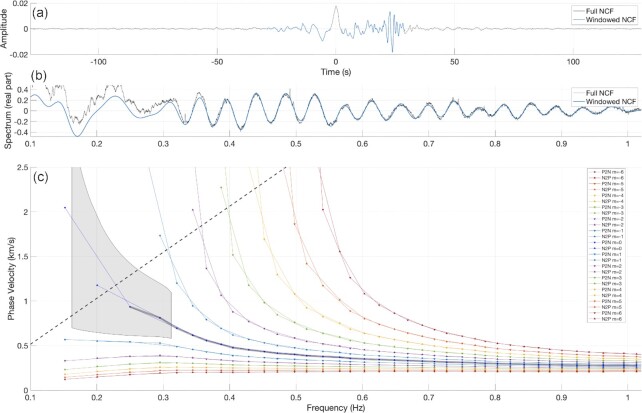
Dispersion curve extraction for the JKA15–JKA17 station pair. (a) NCF for full trace (black) and the signal window (blue). (b) Real part of the spectrum for full trace (black) and the signal window (blue). (c) Phase velocity dispersion curves obtained by fitting the zeros of the Bessel function to the spectrum of the signal window, including alternative curves for different ‘m’ in eq. (3). Grey shading area is one cycle (up and down) around the average dispersion curve from 0.16 to 0.32 Hz. Grey line presents the dispersion curve, which is manually picked.

Fig. 4 shows a dispersion curve measurement for the station pair JKA15–JKA17. As shown in each of these figures, a strong signal is observed in the chosen window, called the signal window (blue line; Fig. 4a). The window length was selected using the expected minimum and maximum velocity of surface waves (200 m s^−1^ and 2 km s^−1^) in our study region. The spectrum of the signal window (blue line; Fig. 4b) clearly shows Bessel-like oscillations that confidently fit the Bessel function's zeros. Branches of phase velocities (Fig. 4c) are indicated for different ‘m’ in Eq. (3). We manually selected a branch ‘m’ that lies within a range of one cycle (a grey area in Fig. 4c) of the reference dispersion curve at frequencies below 0.32 Hz. At the frequencies above, the curve was manually assessed and picked until 1 Hz by considering its smoothness and continuity. The amplitude of oscillations in the spectrum also remains stable up to 1 Hz.

The same procedure was undertaken for all station pairs in this study. We used the reference dispersion curve of the respective zone where the ray path of the station pair lies, either in the subset-1, subset-2 or all-pairs (Figs S2–S4). Moreover, if the ray path propagates through different zones, we consider the combination of those references as a guide.

**Figure 5.**
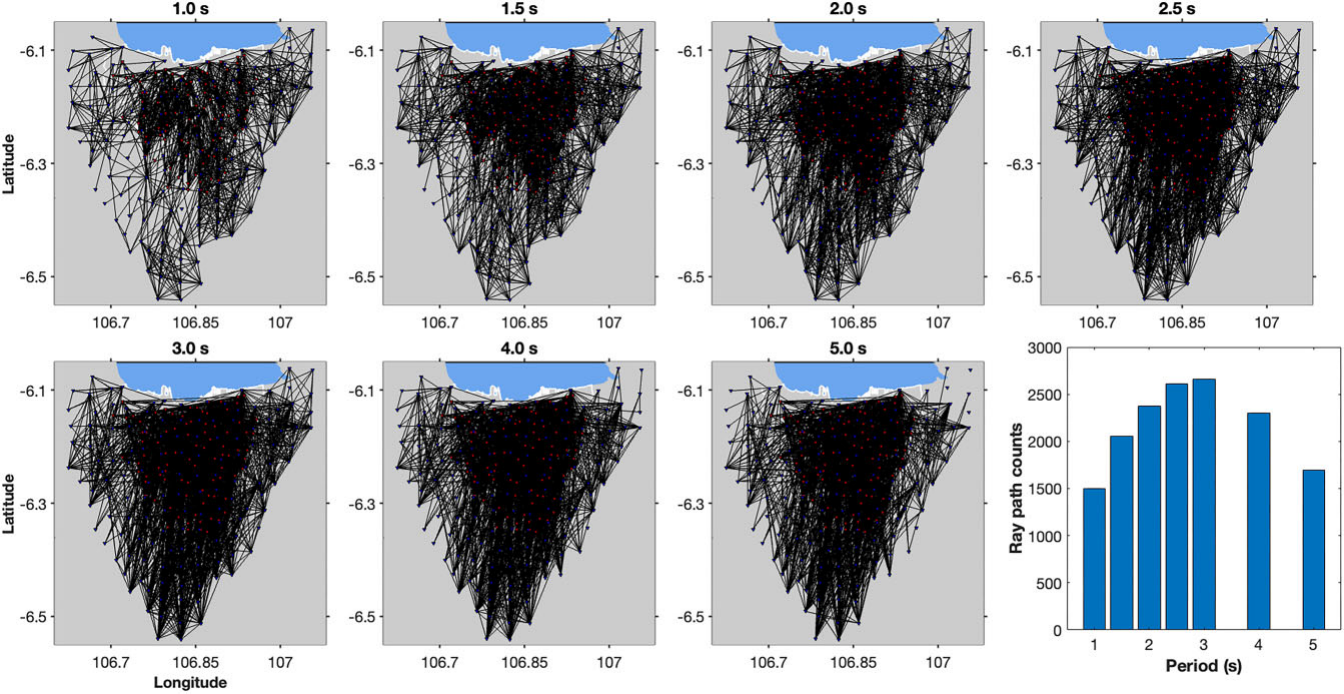
Geometry of straight ray path distributions at selected periods. The bar plot shows the number of ray paths.

We estimated ∼2661 phase velocity dispersion curves collected from NCFs having a high signal-to-noise ratio (SNR). The estimated dispersion curves were used to construct 2-D tomographic maps of Rayleigh wave phase velocity beneath Jakarta for both the 2013–2014 and 2018 networks. We model them in the period domain in which we retrieved their phase velocities at 1, 1.5, 2, 2.5, 3, 4 and 5 s. Fig. 5 depicts the geometry of ray paths and the number of ray paths at the selected periods. The ray paths sample with high density within the city of Jakarta.

### 3.3. Transdimensional Bayesian seismic tomography

We used the Bayesian framework to invert the dispersion curves for maps of Rayleigh wave phase velocity for selected periods. The Bayesian framework combines a probability density of *a priori* realistic Earth model information with a likelihood function as the probability of data to obtain the *posterior* probability distribution function (PDF) for the model given the observed data. In general, Bayes’ theorem is used to express the posterior distribution as:

(4) \begin{eqnarray*}
{\mathrm{posterior\ }} = {\mathrm{\ }}\frac{{{\mathrm{prior\ }} \times {\mathrm{\ likelihood}}}}{{{\mathrm{evidence}}}}
\end{eqnarray*}

In more detail, the conditional probability of the model ${\boldsymbol{m}}$ given the data ${\boldsymbol{d}}$ (the posterior) for model parametrization *k* is defined as:

(5) \begin{eqnarray*}
P\ \left( {{{\boldsymbol{m}}}_k{\mathrm{|}}{\boldsymbol{d}}} \right) = \frac{{P\left( {{\boldsymbol{d}}|{{\boldsymbol{m}}}_k} \right){\mathrm{\ }}P\left( {{{\boldsymbol{m}}}_k} \right)}}{{\smallint P\left( {{\boldsymbol{d}}|{\boldsymbol{m}}{^{\prime}}_k} \right){\mathrm{\ }}d{\mathrm{\ }}{\boldsymbol{m}}{{\mathrm{^{\prime}}}}_k}}\
\end{eqnarray*}

where $P( {{\boldsymbol{d}}|{{\boldsymbol{m}}}_k} )$ is the conditional probability of the data given the model (the likelihood), $P( {{{\boldsymbol{m}}}_k} )$ is the *a priori* probability of model ${{\boldsymbol{m}}}_k$ (the prior), and $\smallint P( {{\boldsymbol{d}}|{\boldsymbol{m}}{{\boldsymbol{^{\prime}}}}_k} )\ d\ {\boldsymbol{m}}{^{\prime}}_k$ is the probability of the data (the evidence). Because the evidence integral appears as a normalization constant in Bayesian inversion, it can be ignored in the probabilistic sampling.

Green (1995) showed that Bayes’ theorem can be used to express a Bayesian hierarchical model to include a set of different model parametrizations represented by the index *k*:

(6) \begin{eqnarray*}
P\ \left( {k,{{\boldsymbol{m}}}_k{\mathrm{|}}{\boldsymbol{d}}} \right) = \frac{{P\left( {{\boldsymbol{d}}|{{\boldsymbol{m}}}_k} \right){\mathrm{\ }}P\left( k \right){\mathrm{\ }}P\left( {{{\boldsymbol{m}}}_k} \right)}}{{\mathop \sum \nolimits_{k{\mathrm{^{\prime}}}} {\smallint }_{{\boldsymbol{m}}{{\mathrm{^{\prime}}}}_{k{\mathrm{^{\prime}}}}}P\left( {{\boldsymbol{d}}|{{\boldsymbol{m}}}_k} \right){\mathrm{\ }}d{\mathrm{\ }}{\boldsymbol{m}}{{\mathrm{^{\prime}}}}_k}}\
\end{eqnarray*}

As with eq. (5), the denominator is regarded as a normalization constant that can be ignored, and the posterior $P( {k,{{\boldsymbol{m}}}_k{\mathrm{|}}{\boldsymbol{d}}} )$ can be explored using the Markov chain Monte Carlo (McMC) algorithm to sample the numerator (Malinverno 2002). This ‘transdimensional’ (Trans-D) sampling allows model sampling to occur over a number of model parametrizations that in our case corresponds to a variable number of either cell, in the case of inversion for 2-D maps of phase velocity, or layers in the case of inversions for *V_S_* depth profiles.

Several studies have shown the successful application of the Trans-D Bayesian method to solve a wide range of inverse problems in seismology and geophysics: ANT (Bodin *et al*. 2012a), surface wave dispersion and receiver functions (Bodin *et al*. 2012b), microtremor (Dettmer *et al*. 2012), airborne electromagnetic (Hawkins *et al*. 2018), finite fault inversion (Benavente *et al*. 2019), global core–mantle boundary tomography (Mousavi *et al*. 2021), gravity and magnetic (Ghalenoei *et al*. 2022), among others. For ANT, the McMC is typically implemented and developed to model 2-D phase or group velocity variations with a parametrization using Voronoi cells (Bodin & Sambridge 2009; Bodin *et al*. 2012a). These were used in studies that apply the Trans-D ANT in various regions, both on local and regional scales (Young *et al*. 2013; Zulfakriza *et al*. 2014; Kim *et al*. 2016; Saygin *et al*. 2016; Zheng *et al*. 2017).

In this tomographic study, we applied the Trans-D Bayesian tree method developed by Hawkins & Sambridge (2015) which uses a tree-based wavelet parametrization, which we henceforth refer to as ‘Trans-D tree’. The Trans-D tree offers flexibility, performance, and efficiency for tomographic imaging problems. The wavelet parametrization based on a tree structure allows a hierarchy of coefficients to represent fine-scale heterogeneity in different resolved parts of the model, which can adapt to the observed data (i.e. ray paths).

We worked at each period independently between 1 and 5 s. To begin with, we used linearized inversion using straight rays to converge to an initial solution quickly. The inversion ran for 1000 000 iterations, and the results acted as a reasonable starting point for approximate statistical information that is then used in a non-linear inversion that allows for ray bending.

Next, we used non-linear inversion for updating ray paths at each iteration to better account for the physics of surface wave propagation between stations. For the forward model, the Trans-D tree uses the fast marching method (FMM; Rawlinson & Sambridge 2004a, b) to trace the wave fonts for a given velocity model. As we expect strong lateral heterogeneity for shallow crustal structures beneath Jakarta, the FMM is beneficial. The Markov chain sampling method in the Trans-D tree includes multiple chains and Parallel Tampering to explore the model space better and speed up the convergence (Sambridge 2013). For non-linear inversion, we used two parallel chains at each temperature of three steps and attempted to swap information every 25 iterations. We ran 50 000 Markov chain steps for each chain.

Lastly, we seeded a new parallel non-linear inversion using the previous parallel non-linear solution. We re-ran another non-linear inversion for 50 000 Markov chain steps with identical sampling parameters. Out of those steps, the first 10 000 iterations are the burn-in samples. We discarded the burn-in samples and used the rest of the iterations to compute the mean model. The chain histories were investigated to indicate whether it has achieved convergence. These 3-steps increase the computational time considerably but result in improved recovery of velocity structure.

### 3.4. Transdimensional 1-D shear wave inversion

Finally, dispersion curves at each of a set of regularly spaced gridpoints are assembled from the 2-D Rayleigh wave phase velocity maps and inverted to obtain 1-D *V_S_* depth profiles. For each curve, the sample points over adjacent period values are interpolated. The dispersion curves were inverted using a Trans-D Bayesian framework, with the details of the method given in Bodin *et al*. (2012b) and Dreiling & Tilmann (2019). In concept, the transdimensional Bayesian approach for *V_S_* inversion is equivalent to that used in the tomographic inversion for phase velocity described above but uses a variable number of horizontal, constant velocity layers to parametrize the *V_S_* depth profile, instead of 2-D wavelets to parametrize the lateral variation of phase velocity.

For the inversion, we use an uninformative (i.e. uniform) prior for model parameters, with a depth range for the interfaces from the surface to 4 km, a maximum of 6 layers, and *V_S_* from 0 to 3 km s^−1^. For the forward model, the dispersion curves are computed with the routine from Herrmann (2013). Although dispersion data is most sensitive to *V_S_*, we demonstrate that it is also sensitive to a significant degree to *P*-wave velocity (*V_P_*) and mass density (*ρ*) (see Fig. S8), especially for cases of a shallow crust with low velocity. Therefore, we calculated *V_P_* and *ρ* inside each layer using the *V_S_*-to-*V_P_* and *V_P_*-to-*ρ* scaling relationships (Brocher 2005):

(7) \begin{eqnarray*}
{V}_p = 0.9409 + 2.0947{\mathrm{\ }}{V}_s - {\mathrm{\ }}0.8206{\mathrm{\ }}{V}_s^2 + {\mathrm{\ }}0.2683{\mathrm{\ }}{V}_s^3 - 0.0251{\mathrm{\ }}{V}_s^4 
\end{eqnarray*}

 

(8) \begin{eqnarray*}
{\mathrm{\rho }} = 1.74\,{V}_p^{0.25}
\end{eqnarray*}

These relations are valid for *V_S_* ≤ 4.5 km s^−1^ (eq. 7) and for 1.5 ≤ *V_P_* ≤ 6.1 km s^−1^ (eq. 8).

The inversion was performed with 24 chains to explore multiple independent parameter spaces. Each chain performed 3 million iterations, with a 1:1 ratio for the burn-in and exploration phase. If some chains failed to converge, the chains would be discarded as outliers. The final posterior distributions collect 100 000 models from the exploration phase, excluding outlier chains (Dreiling *et al*. 2020). We took the mean over the final posterior distributions for the preferred *V_S_* depth profile.

## 4. RESULTS

**Figure 6.**
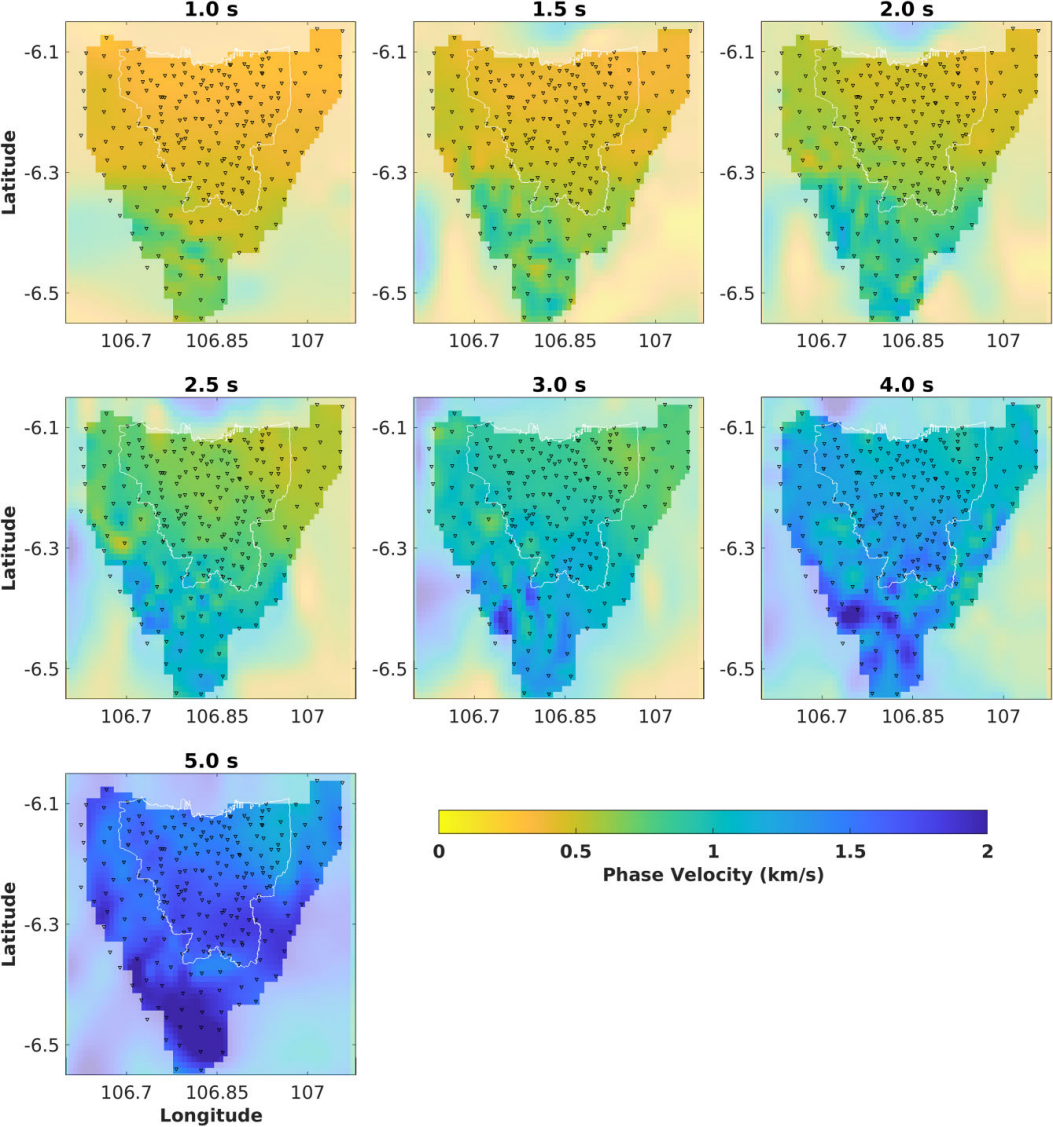
Mean phase velocity tomograms of fundamental-mode Rayleigh wave from Trans-D tree Bayesian inversion for periods from 1 to 5 s. Yellow colour indicates low velocity, while blue colour indicates high velocity.

**Figure 7.**
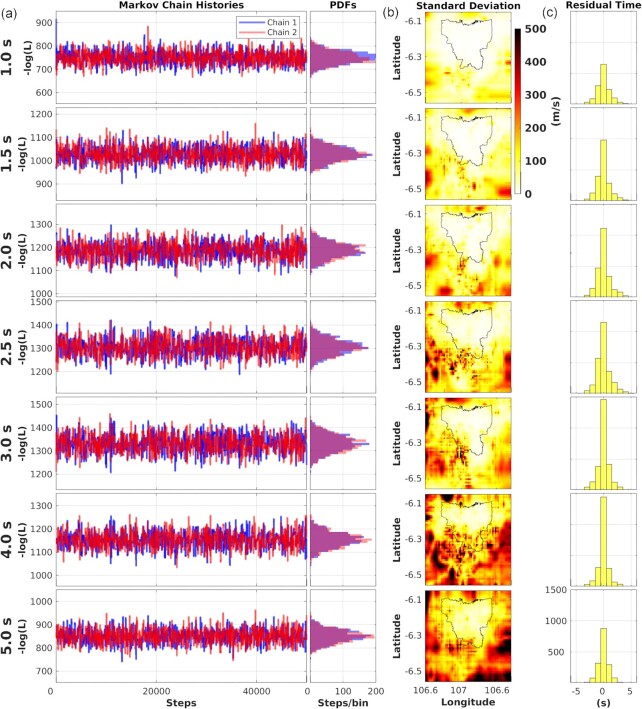
Statistics of tomography results (Fig. 6) from Trans-D tree Bayesian for periods from 1 to 5 s. (a) Markov chain histories for the negative logarithm of the likelihood in the last 50 000 steps. (b) Maps of standard deviation of the tomograms representing uncertainties. (c) Residual histograms of the mean phase velocity tomograms.

Fig. 6 depicts the Rayleigh wave phase velocity maps from periods 1 to 5 s. The corresponding Markov chain histories and uncertainty of each map are given in Fig. 7. The Markov chain histories appear ‘flat’, suggesting a convergence of the Markov chains (Hawkins & Sambridge 2015).

We expect the Rayleigh waves at periods below 2 s to be most sensitive to structure at shallow depth, approximately <1 km, due to the low velocity of sediments. Low velocities of Rayleigh wave (250–500 m s^−1^) appear to cover most of Jakarta, which we presume corresponds to the pervasive shallow sedimentary cover reflected in surface geology maps. The low velocity extends to the western and eastern areas outside of Jakarta, to Tangerang and Bekasi, respectively. Towards the northeast, the low velocity exhibits a more robust anomaly, and it is consistent through longer periods. Meanwhile, toward the south (Depok and Bogor), Rayleigh wave velocities are gradually increasing. This suggests that the depth of the Jakarta Basin decreases southward. South of latitude 6.3°S, a contrast between low and high velocity can be observed. We presume this may relate to the transition of stratigraphy formations. Nevertheless, as shown for longer periods of 4 and 5 s, the velocity coverage seems smooth throughout the study area, suggesting that the deeper structure is more homogenous.

**Figure 8.**
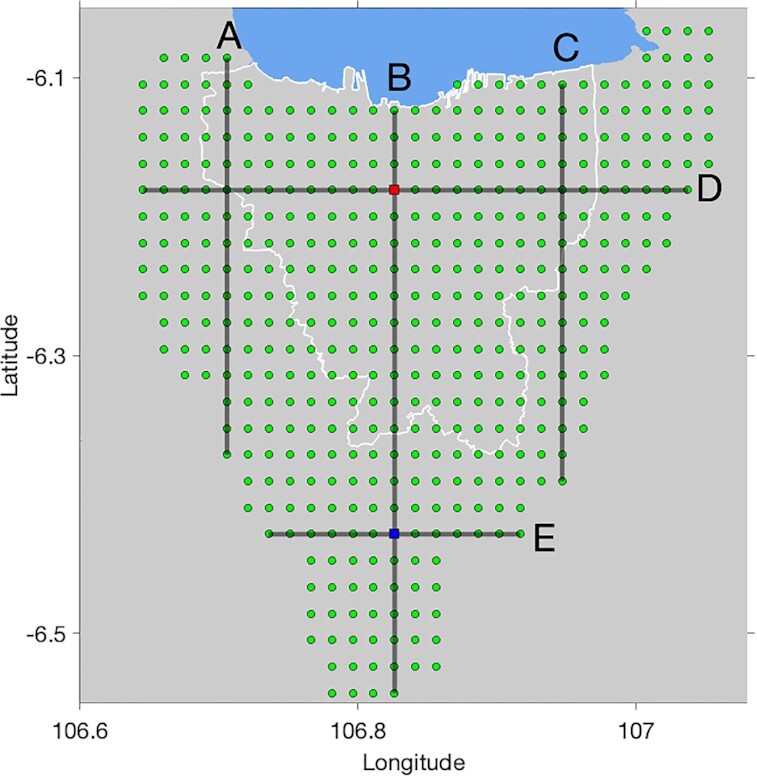
Grid locations (green dots) where dispersion curves are extracted from phase velocity maps and inverted for *V_S_* depth profiles. Blue square is L211 and red square is L224. Black lines A–E represent five selected cross-sections for *V_S_* vertical profiles.

**Figure 9.**
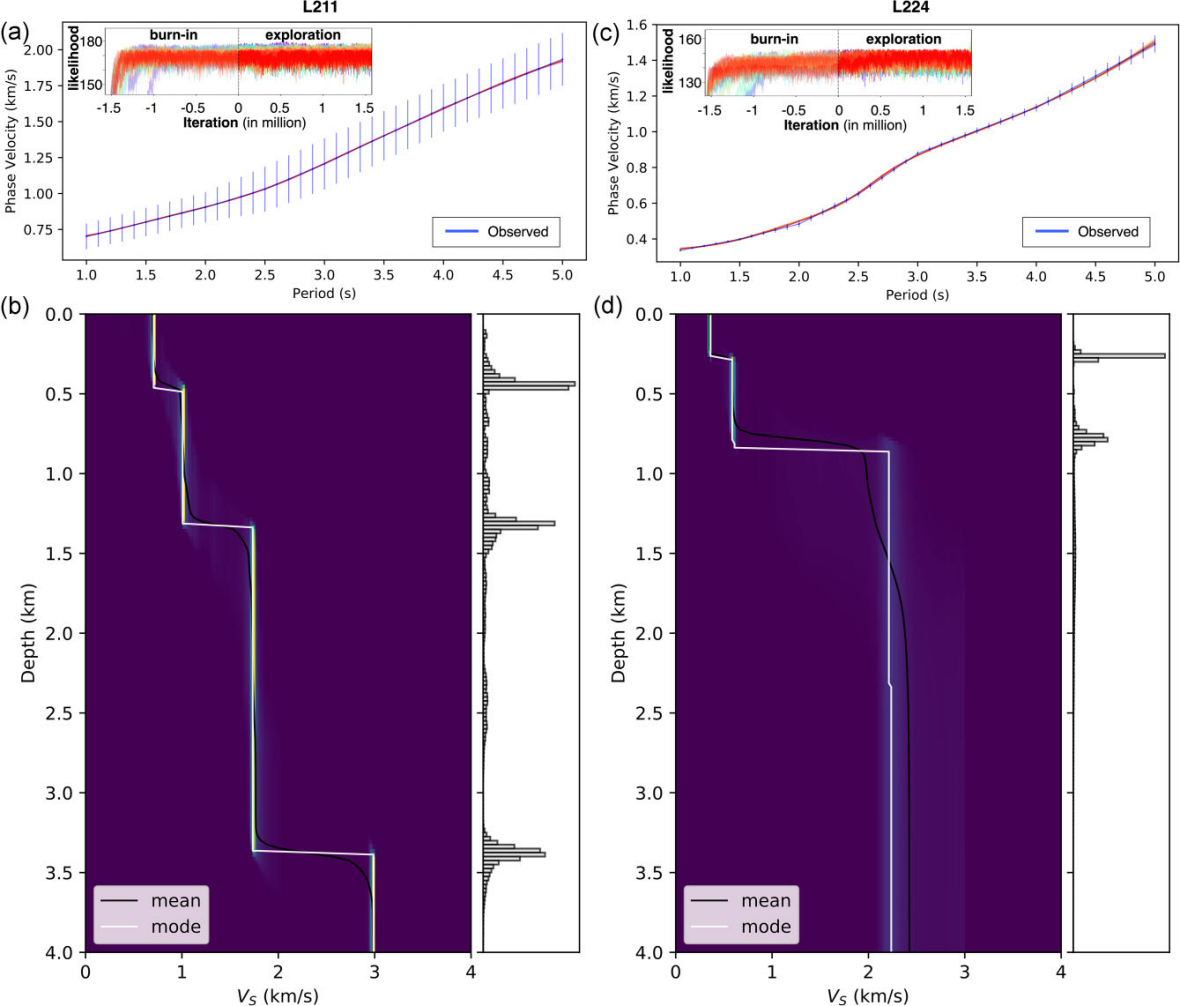
Example of a Trans-D inversion for L211 (a and b; blue square in Fig. 8) and L224 (c and d; red square in Fig. 8). (a) (c) Data fits between observed and calculated curves of 24 chains with their likelihood in 3 million iterations. (b) (d) 1-D *V_S_* posterior distributions (left-hand panel) and interface depth probabilities (right-hand panel).

We extracted over 442 dispersion curves from the phase velocity tomograms at interpolated gridpoints (Fig. 8) with ∼2 km spacing, at which inversions for *V_S_* depth profiles are performed. Fig. 9 gives an example of the inverted dispersion curves for L211 and L224. The quality of data fits for those examples is representative of the other points, in particular showing that the misfit between observed and calculated dispersion curves of 24 chains, in general, is low. The procedure was conducted for all gridpoints in Fig. 8. The *V_S_* profiles (Fig. 9) obtained at each gridpoint were interpolated to constitute a pseudo-3-D model of *V_S_* structure in the Jakarta Basin.

## 5. DISCUSSION

**Figure 10.**
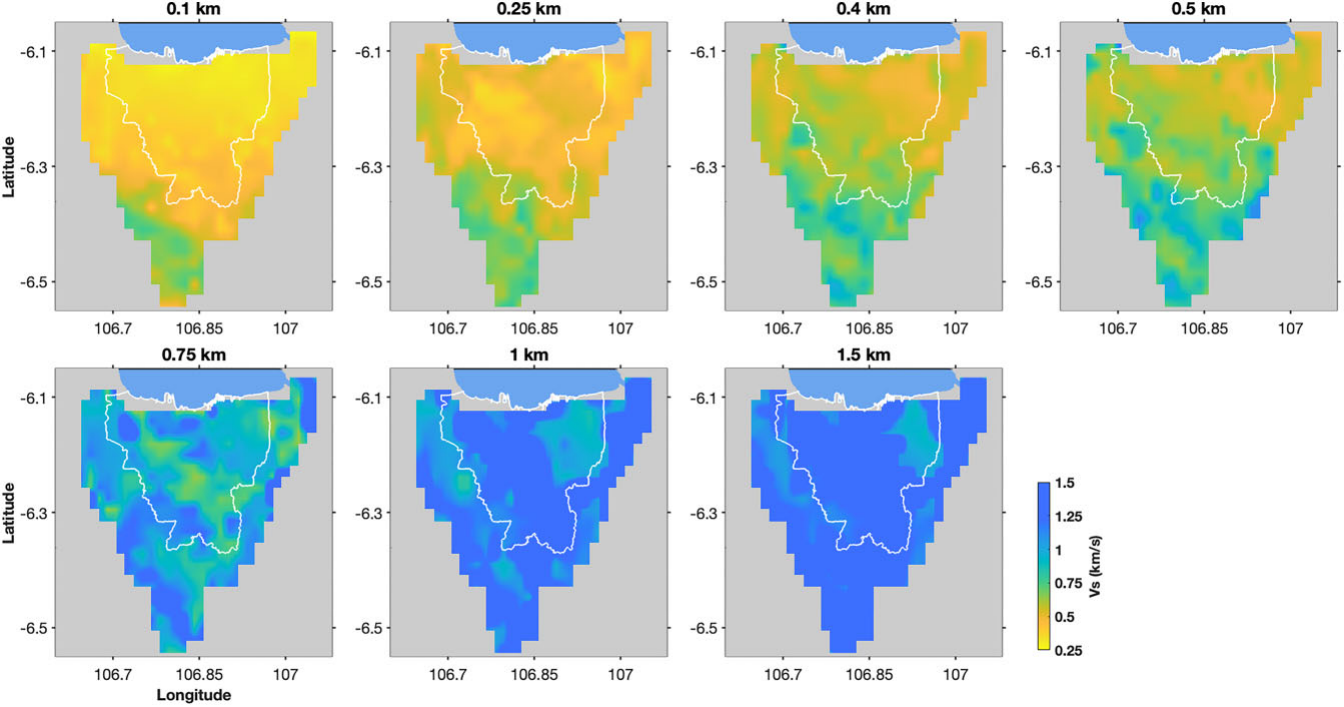
Map slices of *V_S_* across Greater Jakarta from the inversion of phase velocity dispersion curves for depths between 100 m and 1.5 km.

**Figure 11.**
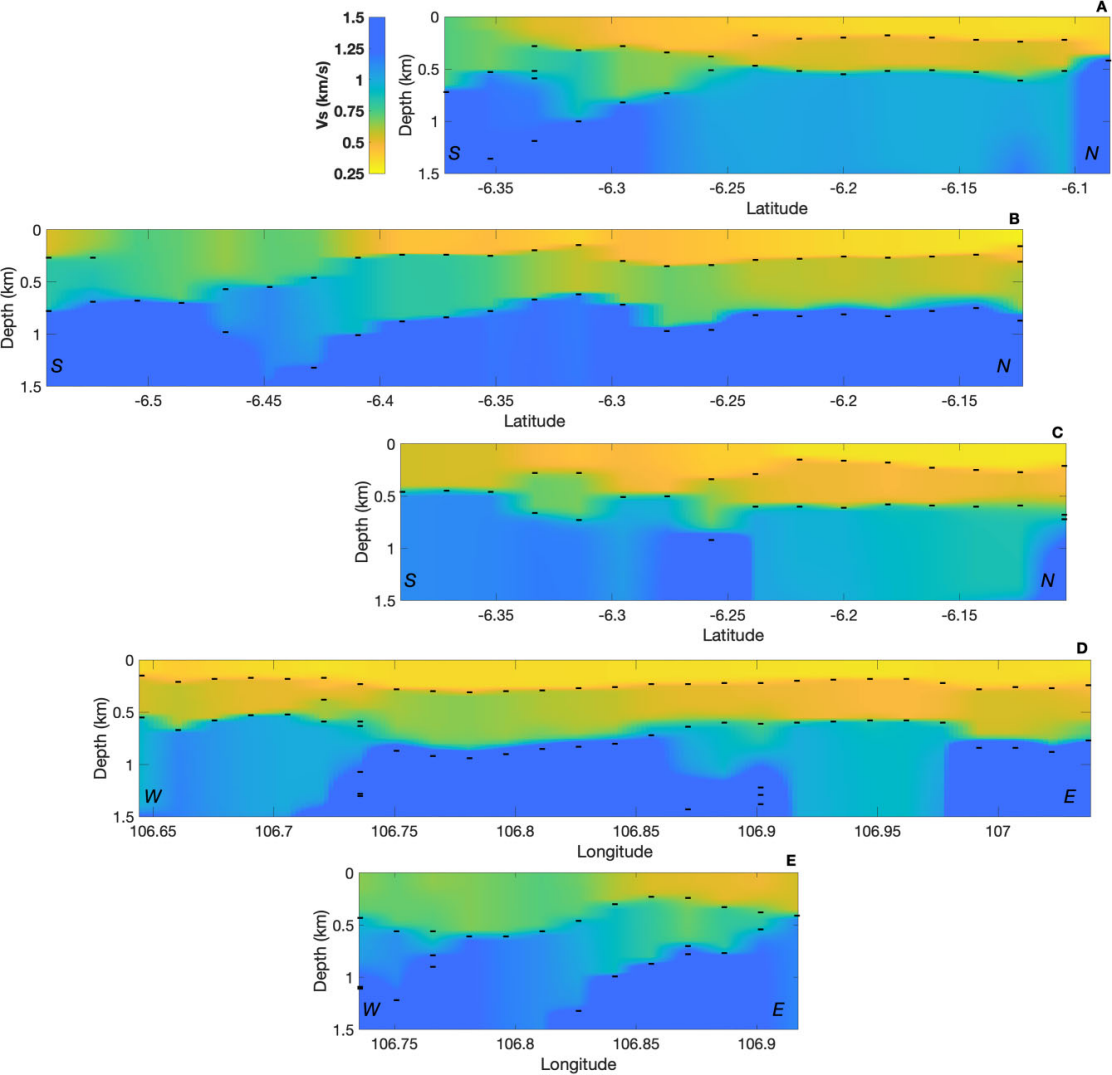
Cross-sections of *V_S_* vertical profiles (A, B, C, D and E) beneath Greater Jakarta for selected five lines shown in Fig. 8. Black horizontal bars represent interfaces depth resulting from Trans-D inversion.

The distribution of the sediment layers in the Jakarta Basin is identified from a pseudo-3-D shear-wave velocity model we developed in this study. Due to the vertical resolution (see Fig. S9), we only interpret the results down to a depth of 1.5 km. The lateral distributions of *V_S_* structure beneath Greater Jakarta between depths 100 m and 1.5 km are shown in Fig. 10, while Fig. 11 depicts the cross-sections of *V_S_* vertical profiles across the area. Fig. 10 shows that in the northern part of Jakarta Basin, the phase velocity inversion in the shallowest layers resolves *V_S_* as low as 250 m s^−1^ (*V_S_* is probably even lower near the surface, in fine-scale layering that is not well resolved (see Cipta *et al*. 2018a; Ridwan *et al*. 2019). *V_S_* as low as 500 m s^−1^ persists to depths as great as 500 m, particularly in the northeast. Farther to the south, the velocity increases more rapidly with depth, suggesting that the southern edge of the basin has been at least partially imaged.

**Figure 12.**
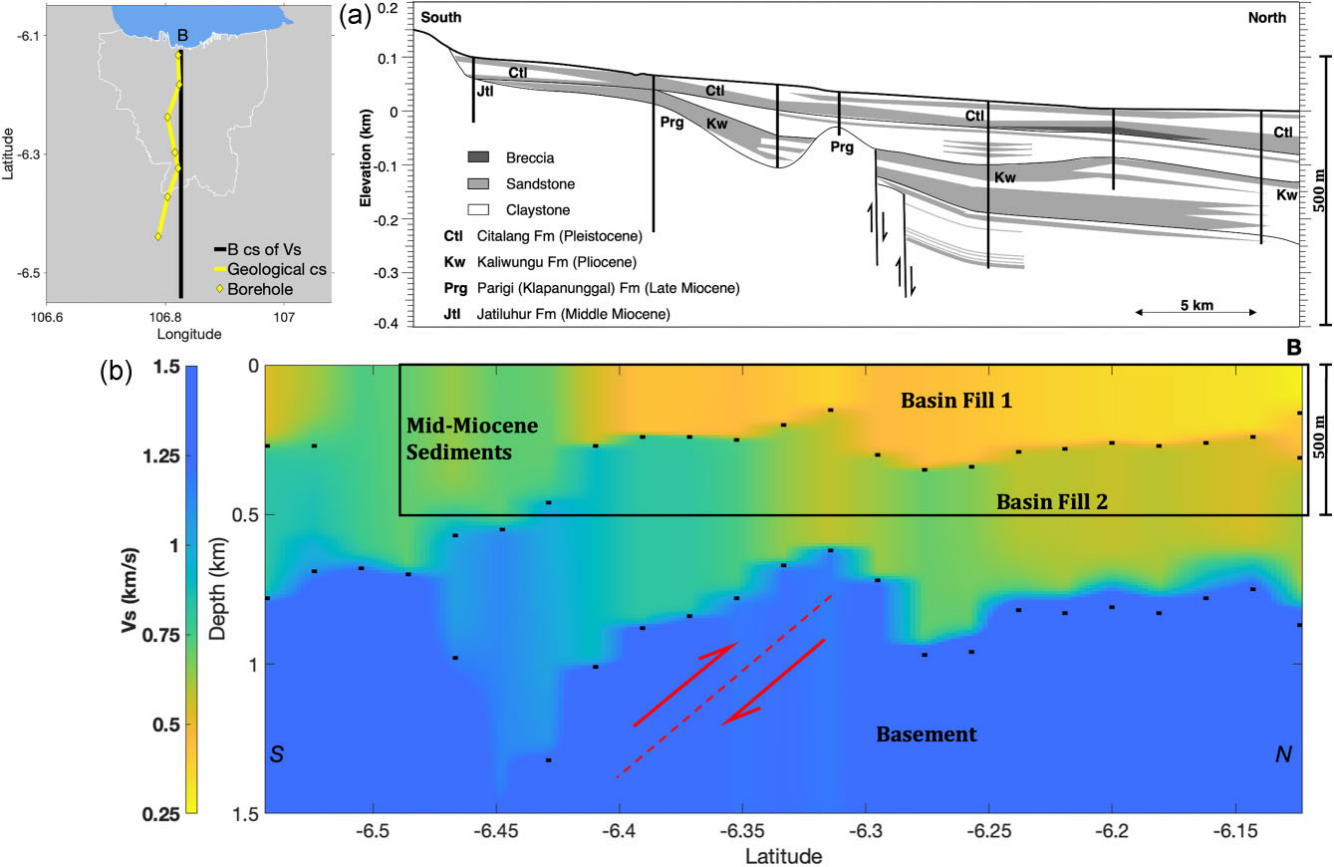
Cross-section (cs) of the Jakarta Basin from south to north. (a) Geological cs derived from borehole data given in Fachri *et al*. (2002) (modified from Fig. 6b). (b) B cs of *V_S_* model from Fig. 11 and the inferred thrust fault (red-dashed line).

Superposed on the *V_S_* cross sections in Fig. 11 are horizontal bars indicating the depth of peaks in the posterior PDFs for interface depth. The shallowest of these appear to define an approximately continuous surface, everywhere but in the southernmost part of the basin, and appears to divide the basin into a shallow (depth < 300 m) layer with *V_S_* in the interval 250–500 m s^−1^, from a deeper layer (200–750 m depth range) having *V_S_* in the interval 500–800 m s^−1^. In Fig. 12, where we compare the cross-section B with a section from Fachri *et al*. (2002) derived from borehole logs, we have designated these layers as ‘Basin Fill 1″ and ‘Basin Fill 2″, respectively. The peaks in interface probability also appear to define an approximately continuous surface at a depth that varies in the depth range between 750 and 1000 m and coincides with an increase in velocity to 1000 m s^−1^ or greater. We designate this as ‘Basement’ (Fig. 12b).

**Figure 13.**
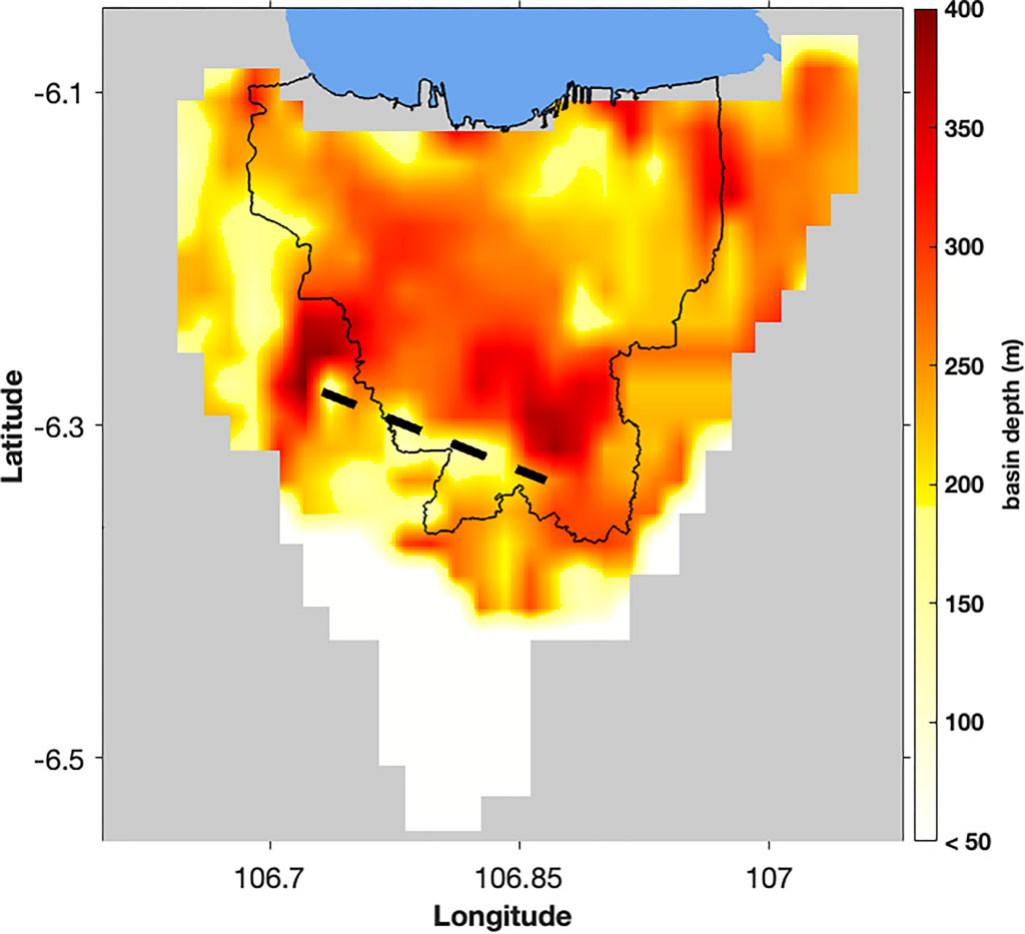
Spatial map of the basal depth of Basin Fill 1 (Pliocene–Pleistocene sediments) and the inferred offset, tentatively interpreted as thrust faulting (black-dashed line).

The base of the layer Basin Fill 1 and its depth variation (Figs 12 and 13) correspond well with the hydrological basement (Fachri *et al*. 2002) interpreted based on borehole data. They identify this interface as the base of Pliocene–Pleistocene sediments that fill the shallow part of much of the Jakarta Basin. The layer we refer to as Basin Fill 2 was identified by Fachri *et al*. (2002) as Late Miocene sediments of low permeability, hence its designation as the hydrological basement. However, none of those boreholes described penetrate much below the Basin Fill 2 layer, and as far as we are aware, no borehole data provide a constraint on the lithology below this layer. We refer to the apparent, abrupt increase in *V_S_* at the base of layer Basin Fill 2 as Basement, having *V_S_* of greater than 1000 m s^−1^. It seems likely that this seismological basement may be associated with widespread carbonate deposition in the Java Sea region that occurred in the early Miocene (Clements & Hall 2007).

Inferred from cross-section B as indicated in Fig. 12(b), *V_S_* in the shallowmost layer gradually shifts from about 500 to 750 m s^−1^ at about latitude −6.4° (see Fig. 13). This seems likely to reflect the shift from Pliocene–Pleistocene sediments of layer Basin Fill 1 to Middle Miocene sediments (having *V_S_* of 750 m s^−1^). We highlight this boundary as the edge of Basin Fill 1, although the very shallow layer of Pleistocene–Holocene alluvium dominating the surface geology in the region (Fachri *et al*. 2002) is unresolvable in this study. Meanwhile, in the southeast (Fig. 11, cross-section E), instead of Basin Fill 1 being replaced by Middle Miocene sediments, cross-section C suggests that layer Basin Fill 2 shallows and replaces layer Basin Fill 1 as the shallowest resolvable layer, which is also consistent with the emergence of Late Miocene sediments near the surface southeast of Jakarta.

Finally, we consider the pronounced offset in both the Basement and the interface between Basin Fill 1 and 2 at around latitude −6.3°. This apparent ‘step’ agrees well with the borehole data of Fachri *et al*. (2002), as indicated in Fig. 12. Although their study seems to imply that this corresponds to normal displacement on a steep, northward dipping, half-graben-like structure, we note that its displacement of the Late Miocene sediments of Basin Fill 2 implies the displacement occurred after extension the Java Sea had ceased in the late Oligocene (Clements & Hall 2007). We offer an alternative interpretation for the basement offset evident in cross section B. We tentatively suggest that the displacement could be the result of thrust movement on a fault dipping southward at a shallower dip, as indicated in Fig. 12(b) and Fig. 13. Such a thrust fault would be consistent with previous suggestions of backarc thrusting in West Java (aka the Baribis Fault) based on geodetic (Koulali *et al*. 2017), geological (Simandjuntak & Barber 1996; Aribowo *et al*. 2022) and seismological (Damanik *et al*. 2021; Widiyantoro *et al*. 2022) data.

There are some limitations in our approach that may lead to ambiguity in our result for the *V_S_* structure of the Jakarta Basin. First, we have interpreted our NCFs as containing only fundamental mode Rayleigh wave energy and our analysis did not account for overtones. Although we have argued above that this is a reasonable interpretation, we have not confidently resolved whether the fundamental mode is always dominant, and indeed in the northern part of the basin where velocities are particularly low and the basement is at considerable depth, there may be overtone energy present which we have not accounted for. This may result in some bias in our model (Rivet *et al*. 2015).

Also, because we used a Bayesian method that uses a constant-velocity layer parametrization, our result for any gridpoint can be regarded as the simplest, layered model that fits the data, and a small uncertainty would imply that this simple, layered model fits the data better than other simple, layered models. It may be that our data are equally well fit by a continuous velocity profile, but from the point of view of the parametrization we use, this would be a more complex model, and the parsimony property of Bayesian inversion (Malinverno 2002) would cause such a model to have less weight. Therefore, based on our data alone, it may be difficult to say with confidence that there is no continuous model that fits the data as well as our simple, layered one. On the other hand, the borehole data of Fachri *et al*. (2002) suggests that the actual velocity structure is indeed layered, and the fact that our result matches the layer depths in the borehole data does give us some confidence that we are retrieving the actual layer depths of the *V_S_* profile in the basin.

## 6. CONCLUSION

We developed a 3-D shear-wave velocity model of the Jakarta Basin covering Jakarta and its adjacent areas of Greater Jakarta. We conducted a 2-stage Trans-D Bayesian inversion of Rayleigh wave dispersion curves derived from seismic noise. First, we applied tomography and constructed 2-D phase velocity maps for periods 1–5 s. Then, we invert these tomographic maps into 1-D depth profiles of shear-wave velocity. And finally, these profiles at gridpoints of ∼2 km spacing were interpolated to constitute a pseudo-3-D *V_S_* model.

The model reveals the Pliocene–Pleistocene sediments gradually thickening from south to north, reaching about 350 m below central Jakarta. The edge of this geologically young basin fill is distinct along the south, being replaced by Middle Miocene sediments. Meanwhile, the designated seismological basement, i.e. the base of the Miocene sediments, is more profound, ranging between 750 and 1000 m. Furthermore, we resolve an apparent offset across south Jakarta, which we tentatively interpret as resulting from thrust displacement on a southward-dipping fault, which may be related to the western segment of the Baribis Fault. While this interpretation is tentative, the potential implications for seismic risk in Jakarta are so substantial that we believe it merits further investigation.

To our knowledge, the earthquake hazard and risk assessments of Greater Jakarta (Irsyam *et al*. 2020) are underestimated and still developing since they account for neither the basin's geometry nor the western Baribis Fault. We recommend further work on a localized seismic hazard assessment for Jakarta to account for these potentially significant effects. The 3-D model of the Jakarta basin we developed in this study is suitable for simulation to appraise basin effects on ground motion during earthquake scenarios and will likely result in seismic amplification (see, e.g. Cipta *et al*. 2018b).

## SUPPORTING INFORMATION

**Figure S1**. Properties of NCFs recorded in the Jakarta Basin. (a) Record section of NCFs referenced to station JK087 in northern Jakarta (see inset Fig. 2a), with the causal part of each NCF corresponding to propagation towards the reference station. Red and green dashed lines show predicted arrival times for surface wave velocities of 1.5 and 0.6 km s^−1^ in the acausal and causal parts of the NCFs, respectively. Grey traces show the original NCFs, while black shows NCFs tapered near lag time 0 sec to reduce noise in the calculation of spectra. (b) Amplitude spectra of the acausal (left-hand panel) and causal (right-hand panel) parts of the NCFs in (a), with grey curves showing single-trace spectra, and green curves showing the average of the single-trace spectra.

**Figure S2**. Synthetic record sections of Rayleigh waves including (a) the fundamental mode only and (b) the fundamental + 1^st^ overtone. The seismograms have been filtered to exclude frequencies less than 0.40 Hz and greater than 0.50 Hz for the acausal and causal parts, respectively. Red and green dashed lines show predicted arrival times for surface wave velocities of 1.5 and 0.6 km s^−1^ in the acausal and causal parts of the NCFs, respectively. (c) Spectra of fundamental and 1st overtone Rayleigh waves, where spectral amplitude have been calculated for a surface point source (Harkrider & Anderson 1966), as well as phase velocities of fundamental and 1st overtone. All synthetics and spectra have been calculated for a *V_S_* profile of the Jakarta Basin model near the centre of the stations used for the record section illustrated in Figure S1.

**Figure S3**. Classification of zones and the seismic stations examined for: (a) all-pairs, (b) subset-1 and (c) subset-2. Blue triangles are the seismic stations deployed between October 2013 and February 2014. Red triangles are the seismic stations deployed between April 2018 and October 2018.

**Figure S4**. Average dispersion curve for all-pairs. (a) Observed real part of NCFs spectrum binned in the frequency–distance domain. (b) Phase velocity dispersion curve by fitting the observed coherency and the Bessel function. Dots show the best fits of phase velocity as a frequency function. (c) 1-D *V_S_* posterior distributions and interface depth probabilities. The light-red line in (b) emphasizes the average dispersion curve between 0.07 and 0.32 Hz calculated from the 1-D *V_S_* model in (c).

**Figure S5**. Average dispersion curve for subset-1. (a) Observed real part of NCFs spectrum binned in the frequency–distance domain. (b) Phase velocity dispersion curve by fitting the observed coherency and the Bessel function. Dots show the best fits of phase velocity as a frequency function. (c) 1-D *V_S_* posterior distributions and interface depth probabilities. The light-red line in (b) emphasizes the average dispersion curve between 0.16 and 0.32 Hz calculated from the 1-D *V_S_* model in (c).

**Figure S6**. Average dispersion curve for subset-2. (a) Observed real part of NCFs spectrum binned in the frequency–distance domain. (b) Phase velocity dispersion curve by fitting the observed coherency and the Bessel function. Dots show the best fits of phase velocity as a frequency function. (c) 1-D *V_S_* posterior distributions and interface depth probabilities. The light-red line in (b) emphasizes the average dispersion curve between 0.18 and 0.36 Hz calculated from the 1-D *V_S_* model in (c).

**Figure S7**. Dispersion curve extraction for the JK050–JK087 station pair. (a) NCF for full trace (black) and the signal window (blue). (b) Real part of the spectrum for full trace (black) and the signal window (blue). (c) Phase velocity dispersion curves obtained by fitting the zeros of the Bessel function to the spectrum of the signal window, including alternative curves for different ‘m’ in eq. (3). Grey shading area is one cycle (up and down) around the average dispersion curve obtained in Fig. S2b from 0.07 to 0.32 Hz. Grey line presents the dispersion curve, which is manually picked.

**Figure S8**. Dispersion curves for Rayleigh wave phase velocity. (a) Shear-wave velocity model and corresponding *V_P_* based on the *V_S_*-to-*V_P_* relationships. (b) Forward models of dispersion curve with the *V_S_*-to-*V_P_* eq. (7) scaling relationship and constants *V_P_/V_S_*. (c) Forward models of dispersion curve with the *V_P_*-to-*ρ* eq. (8) scaling relationship and a constant *ρ*.

**Figure S9**. (a) 1-D velocity model representing the study area. Sensitivity kernels for phase velocity to *V_S_* (b) and layer thickness (c) at periods 1–5 s.

**Figure S10**. Same as Fig. 12(b), but the dashed square points out 8 synthetic models built around the pronounced offset.

**Figure S11**. The true models of *V_S_* depth profiles (top panel) and their forward models of Rayleigh wave phase velocity dispersion curves (bottom panel) for the 8 synthetic models (a–h; models 1–8, respectively).

**Figure S12**. Trans-D inversion for models 5 (a and b) and 7 (c and d). (a) (c) Data fits between observed and calculated curves of 24 chains. (b) (d) 1-D *V_S_* posterior distributions (left) and interface depth probabilities (right).

**Figure S13**. Results of the inversion for the 8 synthetic models (a–h; models 1–8, respectively). Black lines present the true models and blue lines present the inverted models.

**Figure S14**. Statistics of tomography results (Fig. 6) from Trans-D tree Bayesian for periods from 1 to 5 s. (a) Chain histories for the number of wavelet coefficients in the last 50 000 steps. (b) Chain histories for the hierarchical scaling term lambda in the last 50 000 steps.

**Figure S15**. (a) Lines F–J of selected cross-sections for *V_S_* vertical profiles. (b) Cross-sections of *V_S_* vertical profiles (F, G, H, I and J). Black horizontal bars represent interface depth.

Please note: Oxford University Press is not responsible for the content or functionality of any supporting materials supplied by the authors. Any queries (other than missing material) should be directed to the corresponding author for the paper.

## Supplementary Material

ggad176_Supplementary_revFeb23

## Data Availability

The pseudo-3-D shear-wave velocity model of the Jakarta Basin obtained in this study is available via GitHub (https://github.com/rexhavry/Vs-Jakarta-Basin). The other data that support the findings of this study are available from the corresponding author, RVR, upon reasonable request.
