# Supplementary material for: 3-D shallow shear velocity structure of the Jakarta Basin from transdimensional ambient noise tomography
Source: Geophys J Int. 2023 May 3;234(3):1916–32. doi: 10.1093/gji/ggad176 (PMC10173864; doi:10.1093/gji/ggad176)
Supplement: ggad176_Supplementary_revFeb23 [file ggad176_supplementary_revfeb23.pdf]

3D shallow shear velocity structure of the Jakarta Basin from transdimensional ambient noise tomography

Supplementary Material

Rexha Verdhora Ry[1,2*], Phil R. Cummins[1,3], Babak Hejrani[1,3], Sri Widiyantoro[2,4]

[1]Research School of Earth Sciences, The Australian National University, Australia
[2]Global Geophysics Research Group, Faculty of Mining and Petroleum Engineering, Institut Teknologi Bandung, Indonesia
[3]Geoscience Australia
[4]Faculty of Engineering, Maranatha Christian University, Bandung, Indonesia

(*email: rexha.ry@anu.edu.au)

1. CAUSAL VS. ACAUSAL NCFS AND INFLUENCE OF 1ST OVERTONE

To check that our assumption that both causal and acausal parts of the NCFs are dominated by the Rayleigh wave fundamental mode, as described in the last three paragraphs of Section 3.1, we show in Figure S1 that the acausal vs. causal parts of the NCF have different frequency content, which we presume is caused by anisotropy in the sources of ambient seismic noise. Our interpretation that the fast and slow arrivals in the acausal and causal parts of the NCF, respectively, are due to illumination of the fundamental mode GF by low and high frequency energy is checked in Figure S2, which seeks to establish that the 1st overtone energy predicted by our model is small, so that both causal and acausal parts of the NCF are dominated by the fundamental mode.

We leave the resolution of how much (or how little) the 1st overtone contributes to our NCFs to future work.

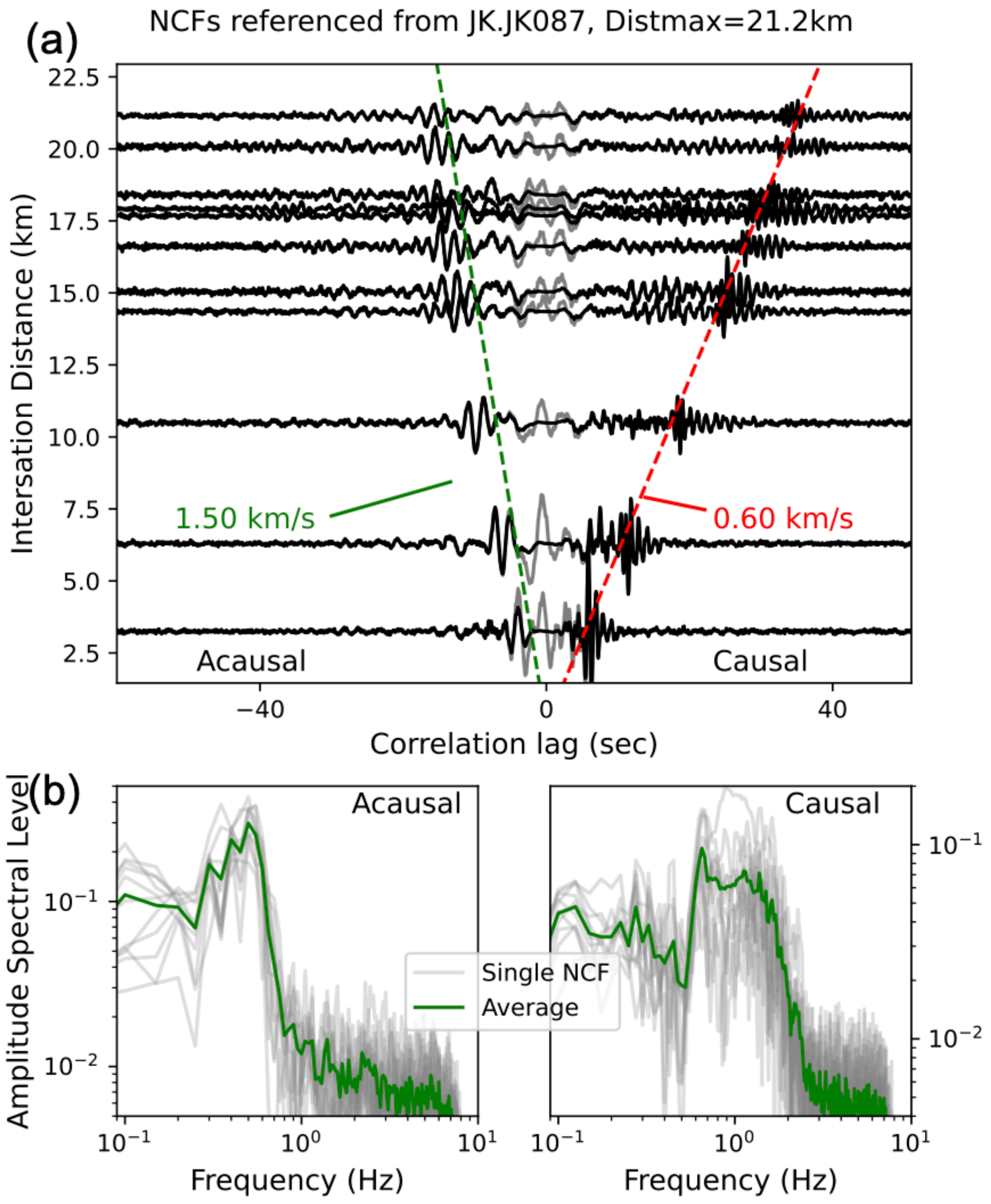

Figure S1. Properties of NCFs recorded in the Jakarta Basin. (a) Record section of NCFs referenced to station JK087 in northern Jakarta (see inset Figure 2a), with the causal part of each NCF corresponding to propagation towards the reference station. Red and green dashed lines show predicted arrival times for surface wave velocities of 1.5 km/s and 0.6 km/s in the acausal and causal parts of the NCFs, respectively. Grey traces show the original NCFs, while black shows NCFs tapered near lag time 0 sec to reduce noise in the calculation of spectra. (b) Amplitude spectra of the acausal (left) and causal (right) parts of the NCFs in (a), with grey curves showing single-trace spectra, and green curves showing the average of the single-trace spectra.

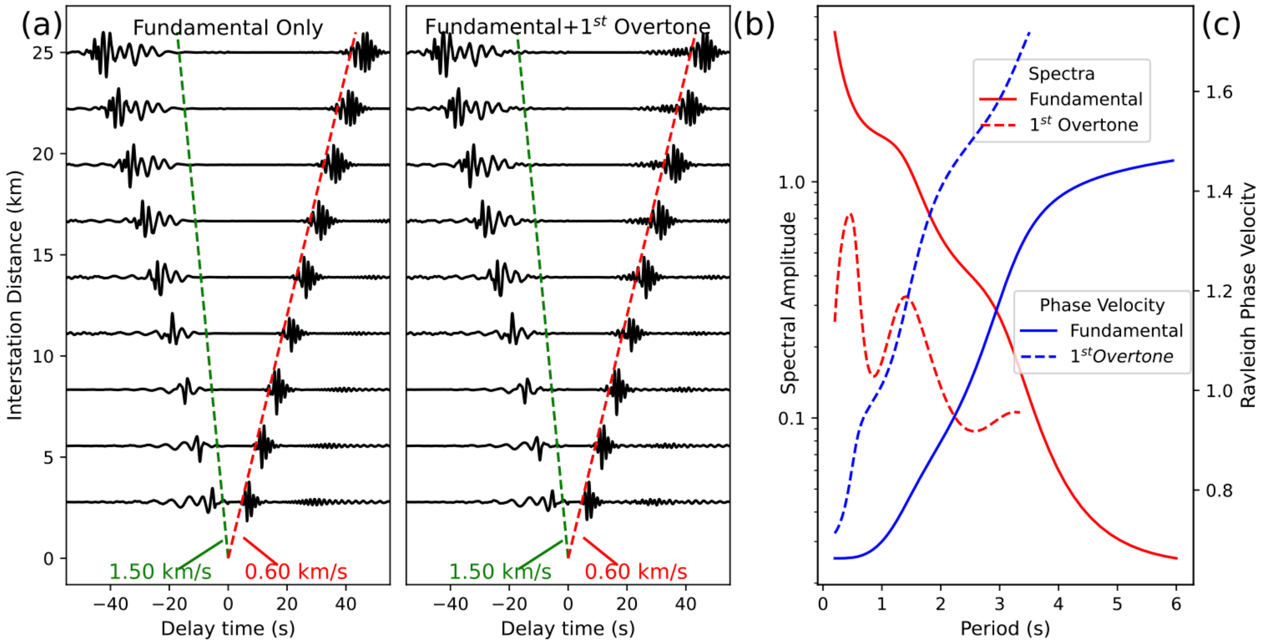

Figure S2. Synthetic record sections of Rayleigh waves including (a) the fundamental mode only and (b) the fundamental + 1st overtone. The seismograms have been filtered to exclude frequencies less than 0.40 Hz and greater than 0.50 Hz for the acausal and causal parts, respectively. Red and green dashed lines show predicted arrival times for surface wave velocities of 1.5 km/s and 0.6 km/s in the acausal and causal parts of the NCFs, respectively. (c) Spectra of fundamental and 1st overtone Rayleigh waves, where spectral amplitude have been calculated for a surface point source (Harkrider and Anderson, 1966), as well as phase velocities of fundamental and 1st overtone. All synthetics and spectra have been calculated for a V_S profile of the Jakarta Basin model near the center of the stations used for the record section illustrated in Figure S1.

2. MODELING AVERAGE DISPERSION CURVES

To better represent the geology in the study area, especially due to changes in the lithology between the south and north basin, we built three average dispersion curves to use as references. The first reference represents the study area in general (all-pairs), while the second and third references represent each zone of the northern part of the study area (subset-1 and subset-2). Figure S3 shows those zones and the stations analyzed separately for each zone. The north area is divided into two

zones that we assumed to be based on surface lithology and different stratigraphy formations in those regions, as shown in Fachri et al. (2002).

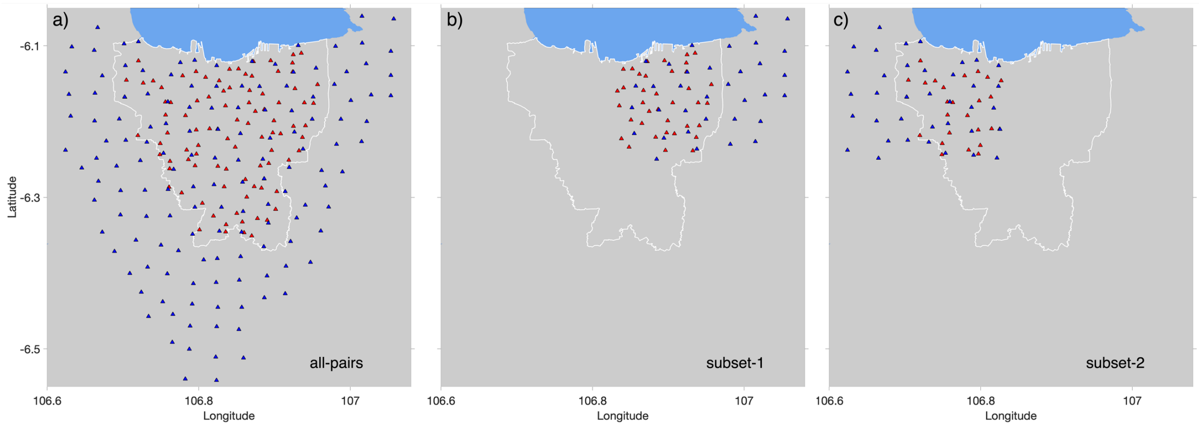

Figure S3. Classification of zones and the seismic stations examined for: (a) all-pairs, (b) subset-1, (c) subset-2. Blue triangles are the seismic stations deployed between October 2013 and February 2014. Red triangles are the seismic stations deployed between April 2018 and October 2018.

To build the first reference, we used NCFs from all station pairs within our seismic network (Figure S3a). The paths between station pairs range from 0 to 45 km (Figure S4a). The grid-search fitting of Eq. 1 recovers reasonable phase velocities between 0.07 and 0.32 Hz (Figure S4b). These phase velocities as a function of frequency reflect an average 1-D shear wave velocity in our study area (Prieto *et al.* 2009). Then, we invert for 1-D V_S profile based on the Bayesian approach (Dreiling & Tilmann 2019) with the model priors: a depth range for the interfaces between 0 and 10 km, a maximum of 6 layers, and V_S from 0 to 4 km/s. We derived V_P and density inside each layer using the V_P-to-V_S (Eq. 7) and V_P-to-density scaling relationships (Eq. 8). Figure S4c shows the posterior distributions of V_S from 24 chains and interface depth probabilities for all-pairs. The interfaces are well defined at ~2 and ~4 km and may relate to the interfaces of Tertiary or Pre-Tertiary rock. Meanwhile, the heterogeneity of shallower crustal structures (<2 km) is simplified in this model.

Next, we built an average dispersion curve for subset-1, using NCFs from station pairs within this sub-array, in the northwestern part of the basin (Figure S3b). The paths between station pairs range from

0 to 24 km (Figure S5a). The grid-search fitting recovers phase velocities between 0.16 and 0.32 Hz (Figure S5b). This defines a phase velocity curve that we invert to obtain an average 1-D V_S model in the subset-1 (Figure S5c). The shallower interface is determined at ~400 m. This shallower structure in the model may relate to younger Tertiary rocks such as Late Miocene formation. Meanwhile, the deeper interfaces are poorly constrained due to limited observations at lower frequencies.

Last, we built an average dispersion curve for subset-2 using NCFs from station pairs within this sub-array (Figure S3c). The paths between station pairs range from 0 to 20 km (Figure S6a). The grid-search fitting recovers phase velocities between 0.18 and 0.36 Hz (Figure S6b). Those points reflect a 1-D V_S model in the subset-2, so we invert for it (Figure S6c). The interfaces are well-defined at ~500 m and ~2 km. The shallower structure is retrieved and may relate to younger Tertiary rocks such as Late Miocene formation. However, compared to the V_S model for all-pairs, the deeper interface (~4 km) is not recovered at all.

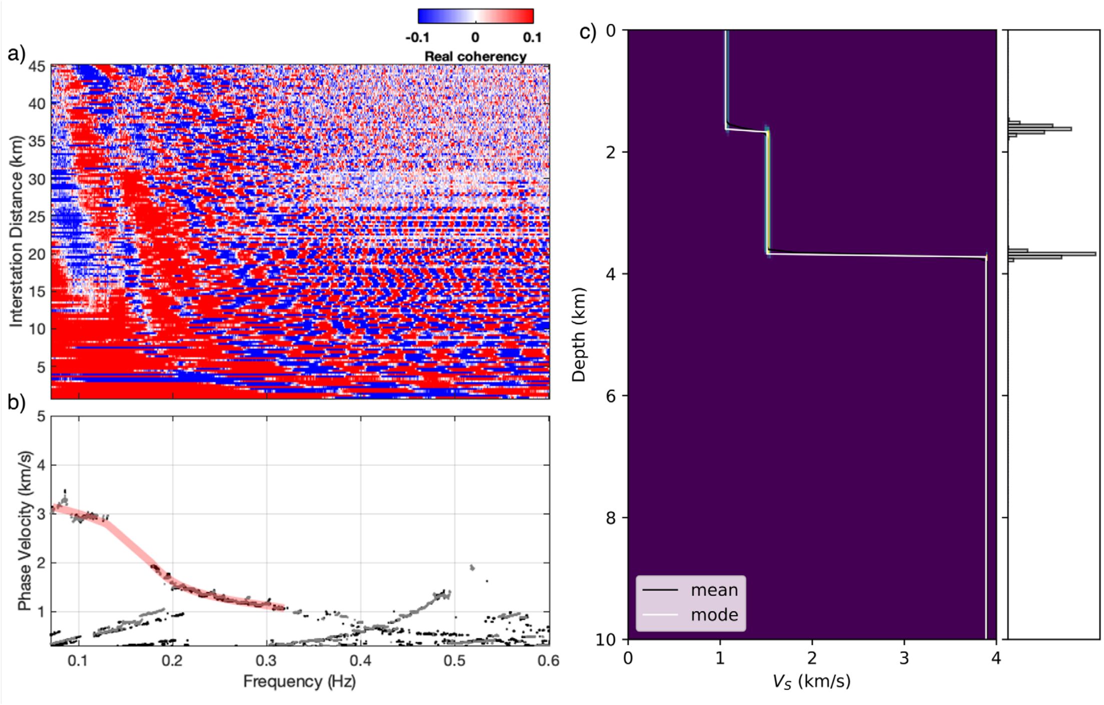

Figure S4. Average dispersion curve for all-pairs. (a) Observed real part of NCFs spectrum binned in the frequency-distance domain. (b) Phase velocity dispersion curve by fitting the observed coherency and the Bessel function. Dots show the best fits of phase velocity as a frequency function. (c) 1-D V_S posterior

distributions and interface depth probabilities. The light-red line in (b) emphasizes the average dispersion curve between 0.07 and 0.32 Hz calculated from the 1-D V_S model in (c).

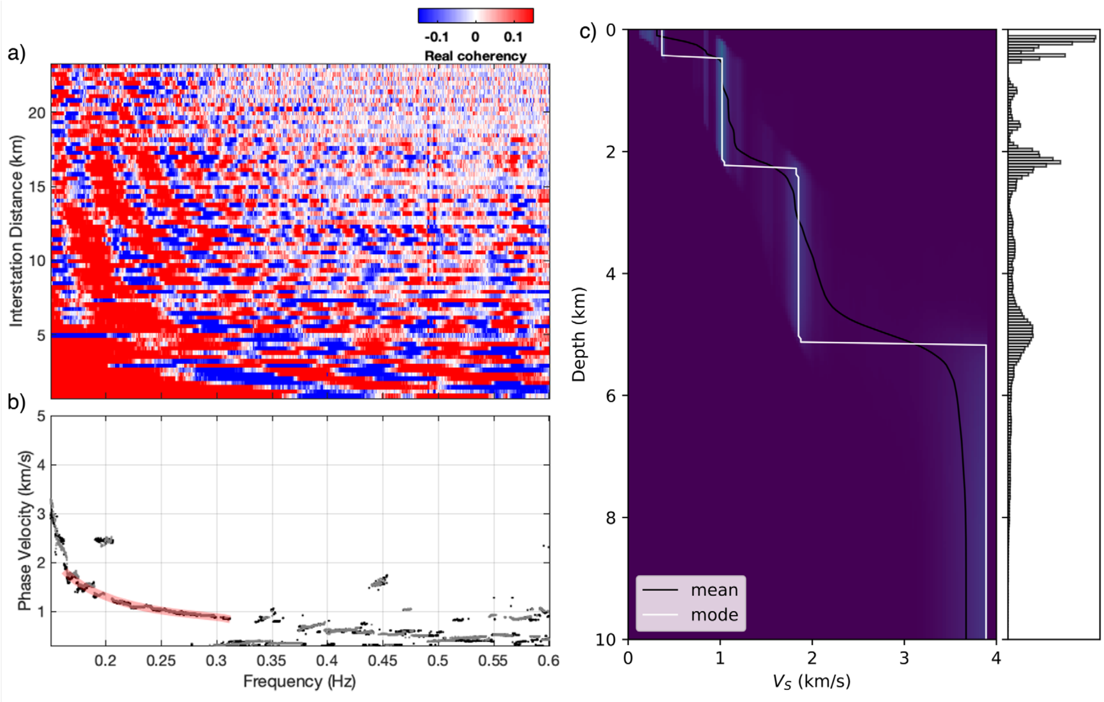

Figure S5. Average dispersion curve for subset-1. (a) Observed real part of NCFs spectrum binned in the frequency-distance domain. (b) Phase velocity dispersion curve by fitting the observed coherency and the Bessel function. Dots show the best fits of phase velocity as a frequency function. (c) 1-D V_S posterior distributions and interface depth probabilities. The light-red line in (b) emphasizes the average dispersion curve between 0.16 and 0.32 Hz calculated from the 1-D V_S model in (c).

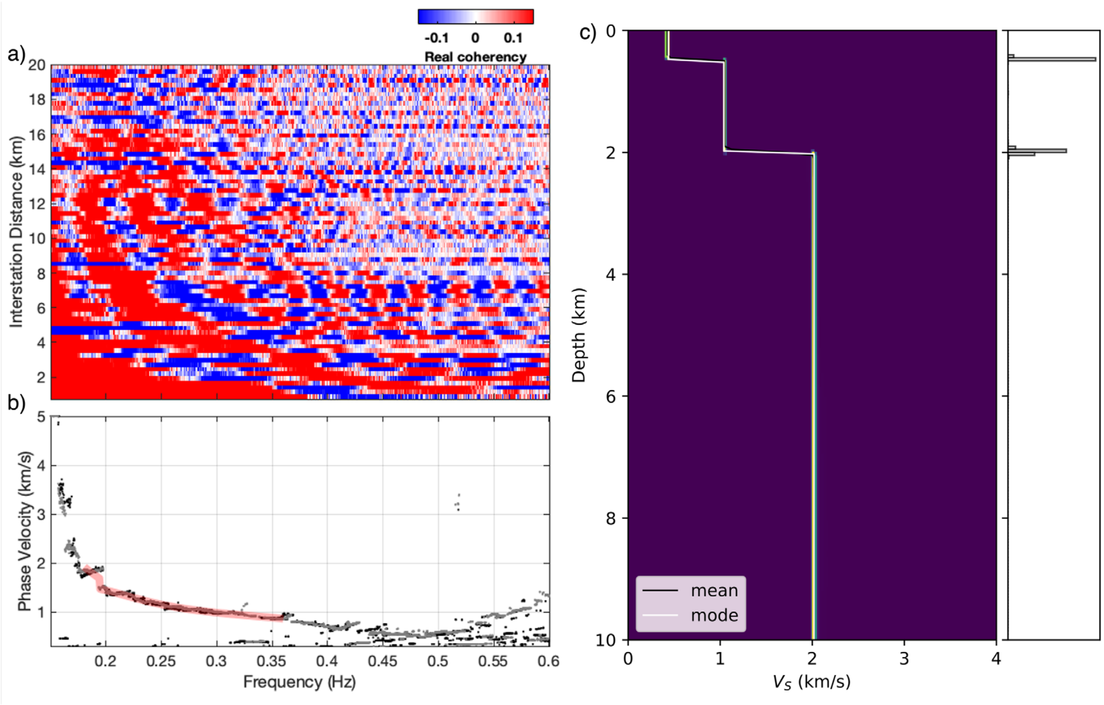

Figure S6. Average dispersion curve for subset-2. (a) Observed real part of NCFs spectrum binned in the frequency-distance domain. (b) Phase velocity dispersion curve by fitting the observed coherency and the Bessel function. Dots show the best fits of phase velocity as a frequency function. (c) 1-D V_S posterior distributions and interface depth probabilities. The light-red line in (b) emphasizes the average dispersion curve between 0.18 and 0.36 Hz calculated from the 1-D V_S model in (c).

Figure S7 shows a dispersion curve measurement for the station pair JK050–JK087. The reference is obtained from the average dispersion curve for all-pairs (Figure S3a & Figure S4). We manually selected a branch ‘m’ that lies within a range of one cycle (a grey area in Figure S7c) of the reference dispersion curve obtained at frequencies below 0.32 Hz. At the frequencies above, the amplitude of oscillations in the spectrum remains stable up to 0.4 Hz, so we manually picked the curve until 0.4. The signal quality of this pair is considerably fair compared to the station pair JKA15–JKA17 (Fig. 4). It is worth noting that most of the NCFs are like either of those.

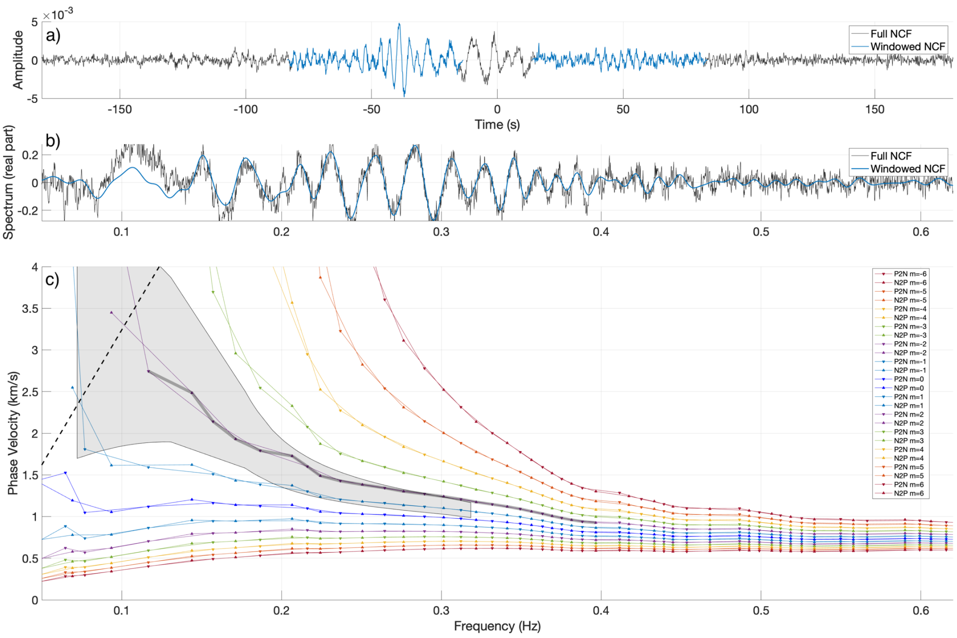

Figure S7. Dispersion curve extraction for the JK050–JK087 station pair. (a) NCF for full trace (black) and the signal window (blue). (b) Real part of the spectrum for full trace (black) and the signal window (blue). (c) Phase velocity dispersion curves obtained by fitting the zeros of the Bessel function to the spectrum of the signal window, including alternative curves for different ‘m’ in Eq. (3). Grey shading area is one cycle (up and down) around the average dispersion curve obtained in Figure S4(b) from 0.07 to 0.32 Hz. Grey line presents the dispersion curve, which is manually picked.

3. FORWARD MODELS OF DISPERSION CURVES

For the forward model (Figure S8), the dispersion curves are computed with the routine from Herrmann (2013). We demonstrate that V_P and ρ significantly contribute to a dispersion curve, especially for the shallow crust and sedimentary cases. Furthermore, a constant V_P/V_S underestimates the calculation in the forward model. Brocher (2005) collected V_P and V_S laboratory measurements for typical lithologies. His results show that for quaternary alluvium, V_S may go in a range of 0.3 – 1 km/s; still, V_P would lie between 1.5 and 2.5 km/s. In other words, the V_P/V_S can be as high as 5 for a layer that has a very low V_S.

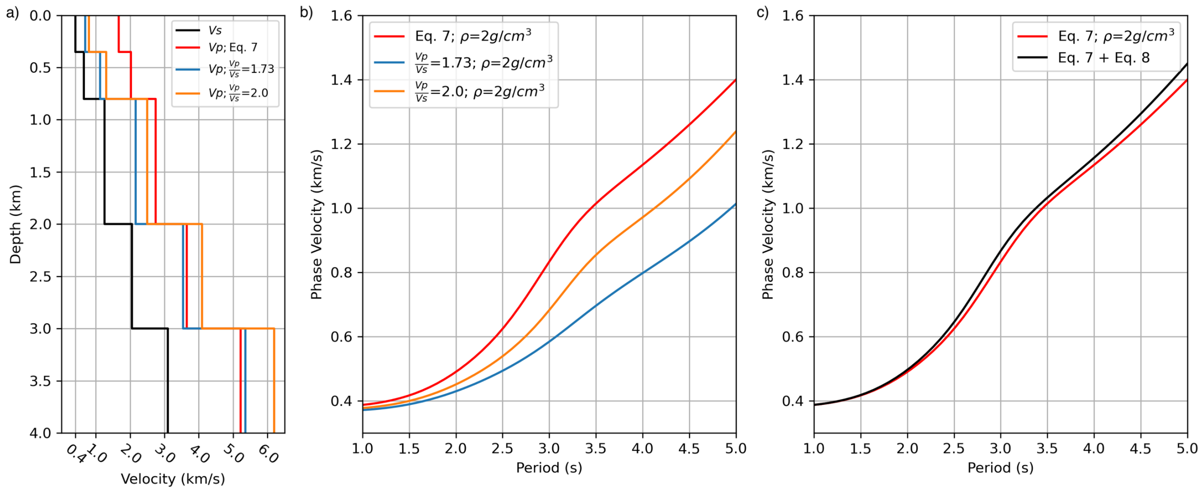

Figure S8. Dispersion curves for Rayleigh wave phase velocity. (a) Shear-wave velocity model and corresponding V_P based on the V_S-to-V_P relationships. (b) Forward models of dispersion curve with the V_S-to-V_P (Eq. 7) scaling relationship and constant V_P/V_S. (c) Forward models of dispersion curve with the V_P-to-ρ (Eq. 8) scaling relationship and a constant ρ.

4. SENSITIVITY KERNELS FOR DEPTH PROFILE

To better understand the vertical resolution of the depth profiles, we calculated sensitivity kernels for phase velocity to V_S and layer thickness. Figure S9 depicts the sensitivity kernels at periods 1 – 5 s using a 1D velocity model representing the study area. These sensitivity kernels show that the period range of our observations is sensitive mainly to the first and second layers, with simply a modest contribution from the third layer at the longest period (5 s). Although we explored the sensitivity to V_S structure as deep as 4 km, the resolution only allows us to recover a depth profile up to 1.5 km due to the limited range of utilized periods. Likewise, the weak sensitivity to the third layer's V_S should be noted cautiously in interpreting the results.

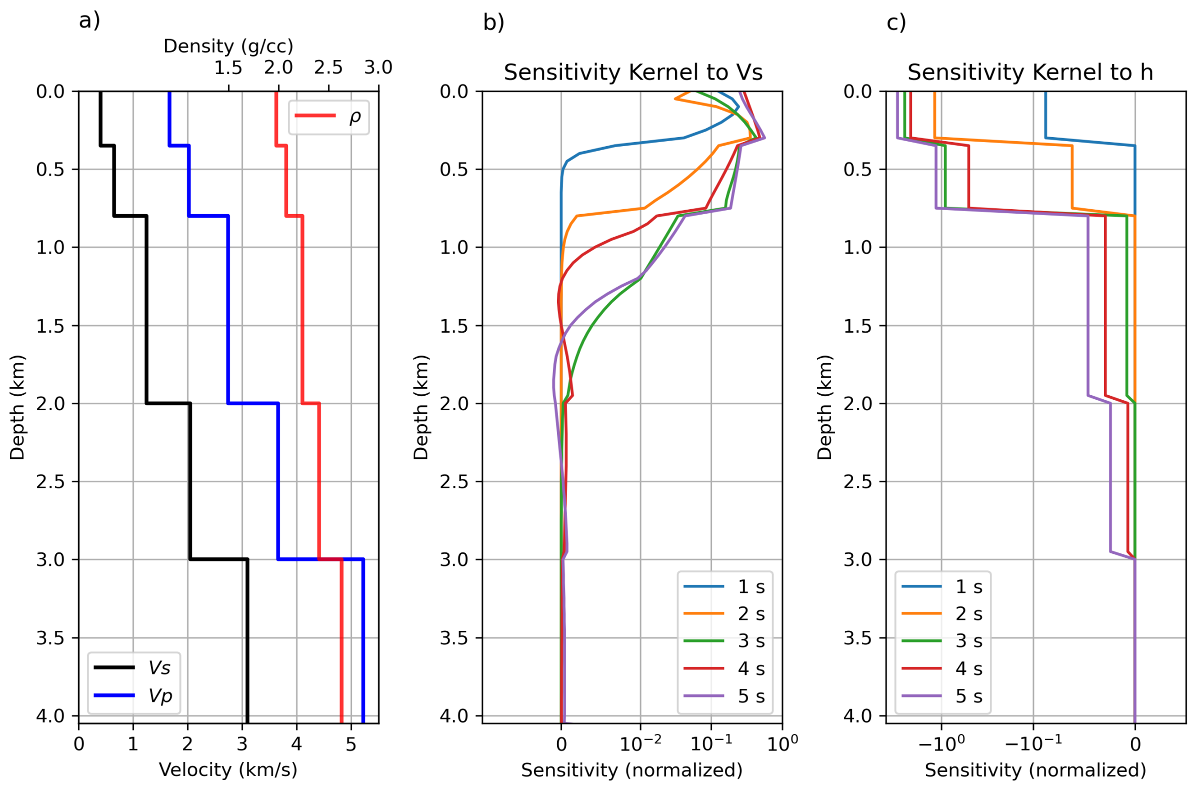

Figure S9. (a) 1D velocity model representing the study area. Sensitivity kernels for phase velocity to V_S (b) and layer thickness (c) at periods 1 – 5 s.

5. SYNTHETIC TEST FOR LAYERED MODELS

We derived the inferred fault given in Fig. 12b based on the offset observed through the depth interfaces of the south-to-north V_S profiles. We used a Bayesian method that uses a constant-velocity layer parameterization, hence, our result for any grid point can be regarded as the simplest, layered model that fits the data. A small uncertainty would imply that this simple, layered model fits the data better than other simple, layered models, which is shown by the probabilities function. Our result matches the layer depths in the borehole data, especially around the pronounced offset of the interface between Basin Fill 1 and 2 at around latitude -6.3°.

To observe the capability of the inversion in retrieving the depth interfaces of layered models, we conducted a synthetic test. We built 8 synthetic models around the pronounced offset (Figure S10).

We extracted the V_S depth profiles (Figure S11; top panel), called the true models. Then, we calculated their forward models of Rayleigh wave phase velocity dispersion curves (Figure S11; bottom panel) that create the synthetic observed data from known V_S models (the true models). The inversion is conducted on this synthetic observed data to inspect whether we can recover the true models or not.

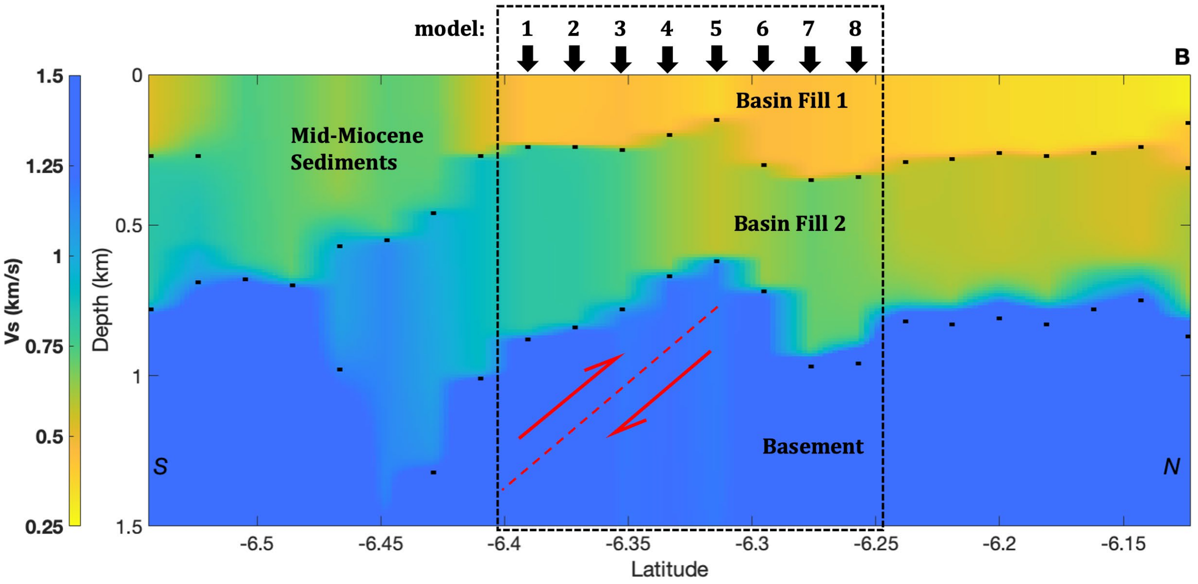

Figure S10. Same as Fig. 12b, but the dashed square points out 8 synthetic models built around the pronounced offset.

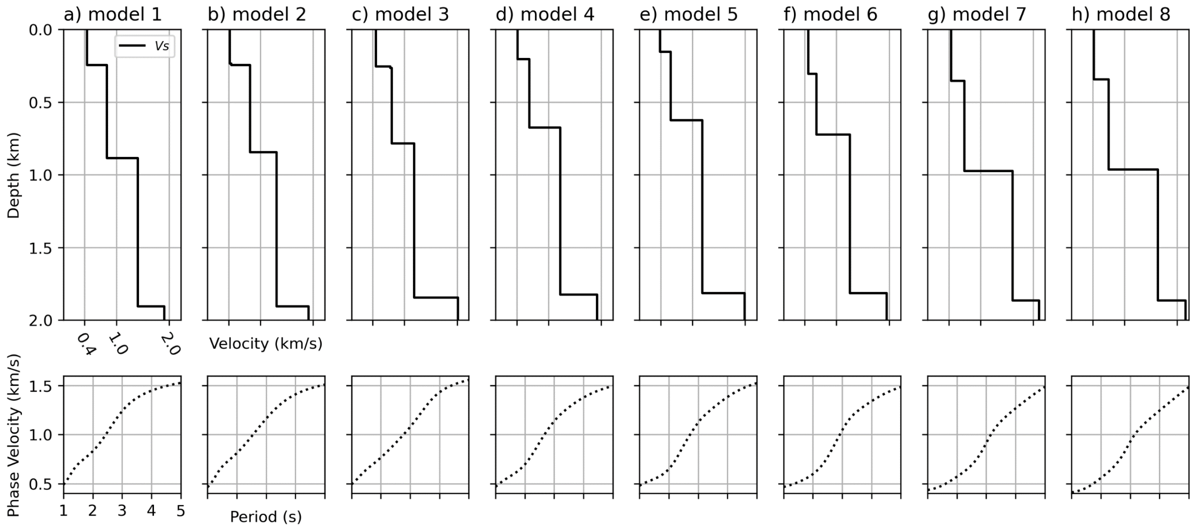

Figure S11. The true models of V_S depth profiles (top) and their forward models of Rayleigh wave phase velocity dispersion curves (bottom) for the 8 synthetic models (a – h; models 1 – 8, respectively).

For the inversion, we used the same approach as given in Section 3.4. Figure S12 gives an example of the inverted dispersion curves for model 5 and model 7. The calculated dispersion curves of 24 chains fit the observed data very well. Figure S13 shows the results for the 8 synthetic models. It is shown that we were able to recover the true model and retrieve the depth interfaces of layered models with sufficiently low uncertainty. Reflecting on our result given in Fig. 12, this suggests that we are retrieving the actual layer depths of the V_S profile in the basin.

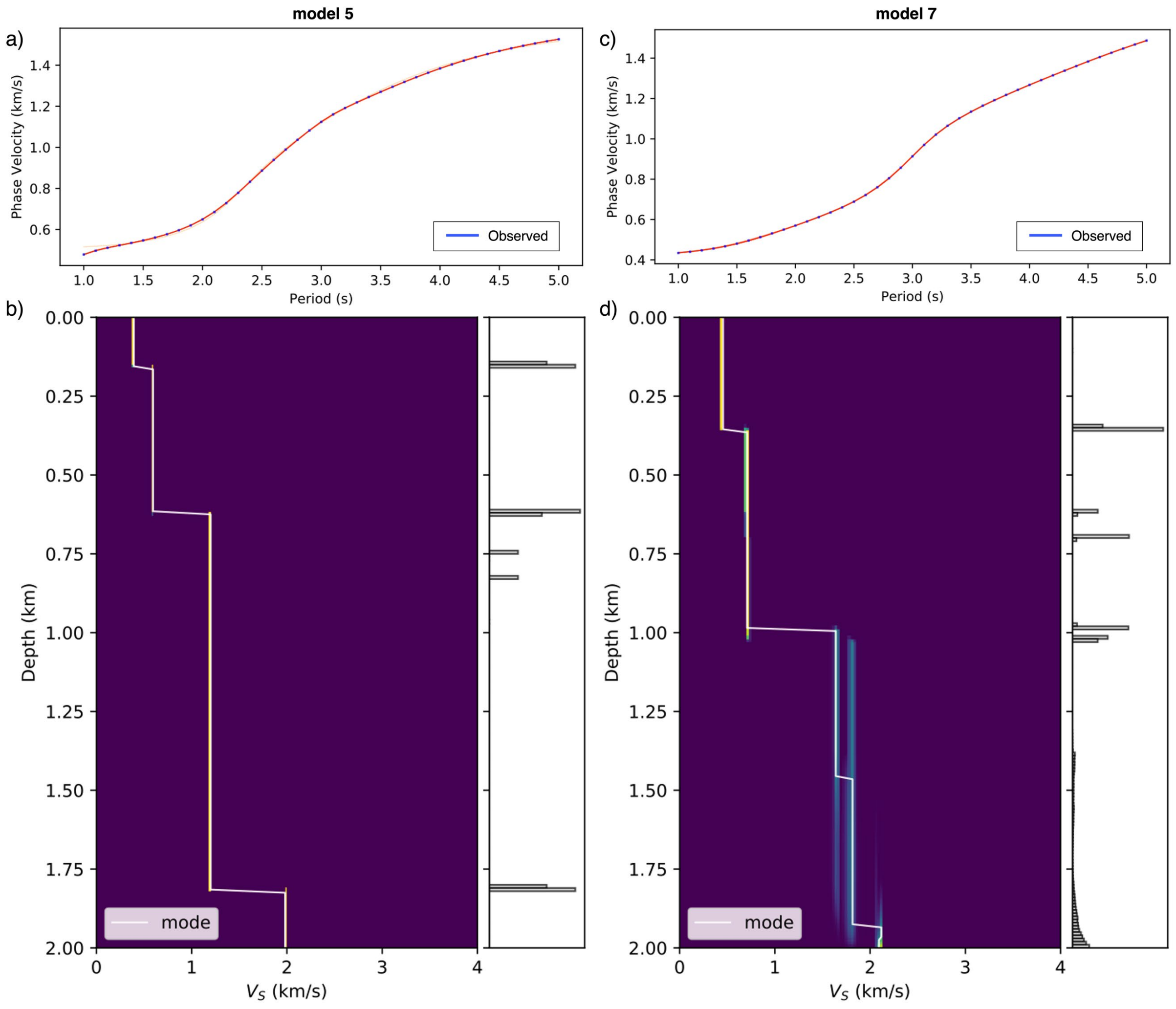

Figure S12. Trans-D inversion for models 5 (a and b) and 7 (c and d). (a) (c) Data fits between observed and calculated curves of 24 chains. (b) (d) 1-D V_S posterior distributions (left) and interface depth probabilities (right).

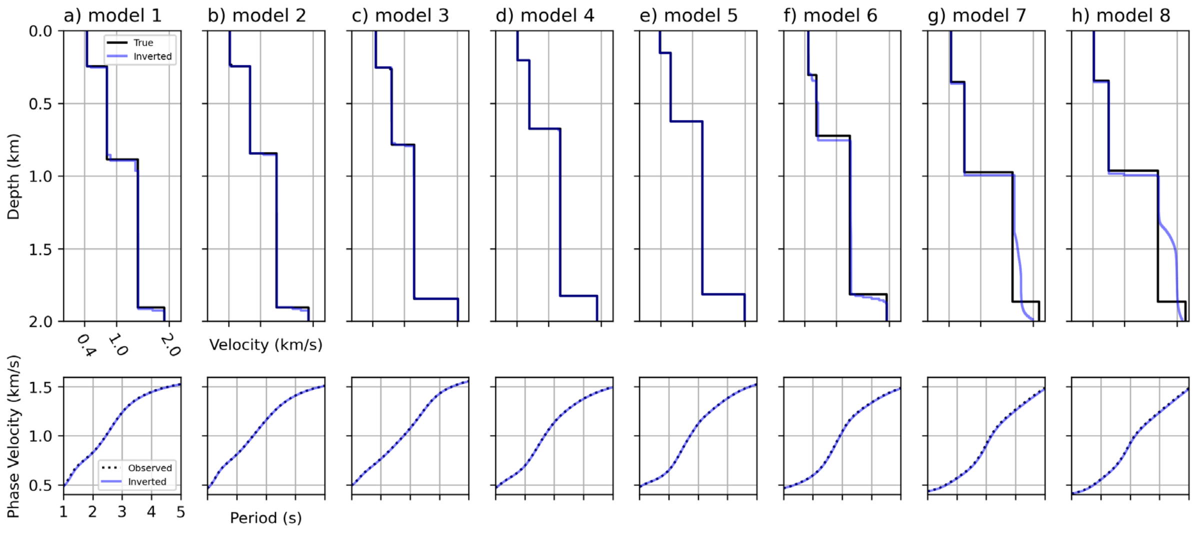

Figure S13. Results of the inversion for the 8 synthetic models (a – h; models 1 – 8, respectively). Black lines present the true models and blue lines present the inverted models.

6. ADDITIONAL FIGURES

Complementary to Section 3.3 and the Rayleigh wave phase velocity maps given in Section 4, the Trans-D tree approach with a wavelet parameterization constructs wavelet coefficients on a model in a hierarchy from coarse to fine length scale to represent an image. The corresponding chain histories of the wavelet parameters for the Rayleigh wave phase velocity maps from periods 1 to 5 s are given in Figure S14. These present the range of complexity allowed in the inversion (level of trees).

Complementary to the discussion in Section 5, the basin edge is distinct along the south, being replaced by Middle Miocene sediments. Figure S15 depicts south-to-north cross-sections parallel to cross-section B (Fig. 11). At the shallowest part of the V_S profiles, farther to the south, the velocity increases more rapidly with depth. This suggests that the southern edge of the basin has been partially revealed.

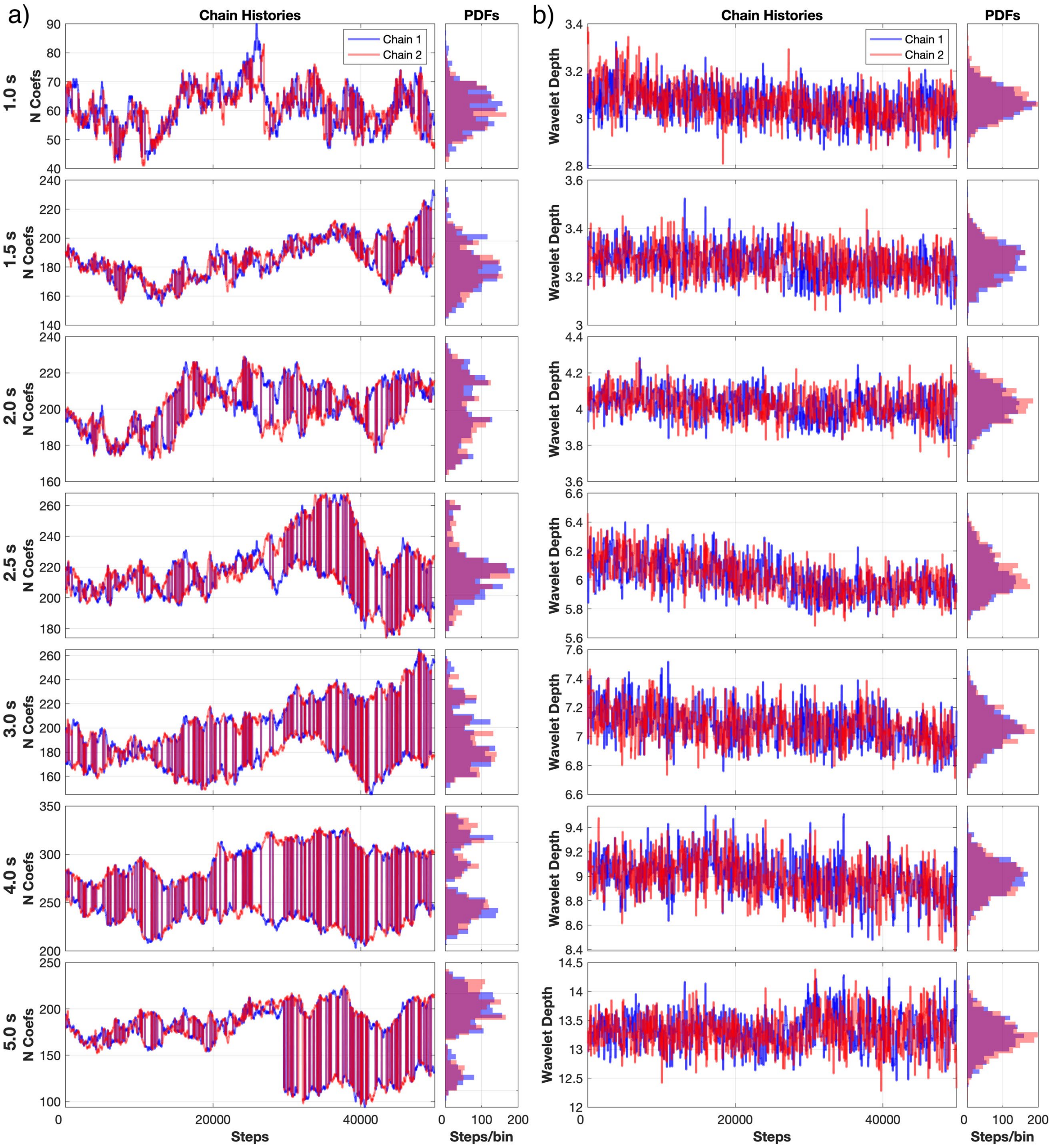

Figure S14. Statistics of tomography results (Fig. 6) from Trans-D tree Bayesian for periods from 1 to 5s. (a) Chain histories for the number of wavelet coefficients in the last 50,000 steps. (b) Chain histories for the hierarchical scaling term lambda in the last 50,000 steps.

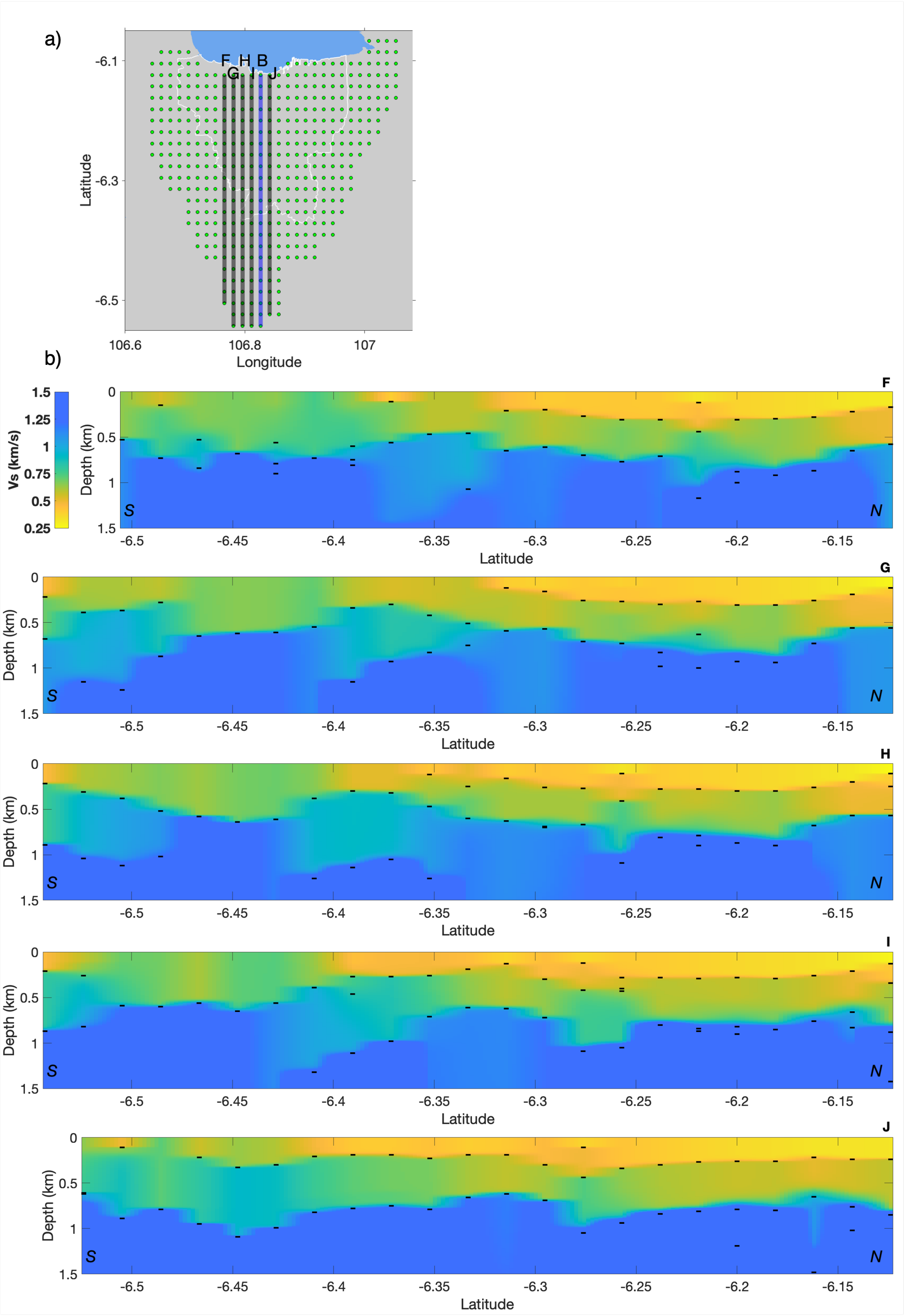

Figure S15. (a) Lines F – J of selected cross-sections for V_S vertical profiles. (b) Cross-sections of V_S vertical profiles (F, G, H, I, and J). Black horizontal bars represent interface depth.
